# Tumour Localisation Technologies in Colorectal Cancer Surgery: A Scoping Review of Marking and Detection Methods

**DOI:** 10.3390/diagnostics16131952

**Published:** 2026-06-23

**Authors:** Mircea Fulea, Mihaela Mocan, Mircea Murar, Bogdan Mocan, Vasile Bințințan

**Affiliations:** 1 Design Engineering and Robotics Department, Technical University of Cluj-Napoca, 400114 Cluj-Napoca, Romania; mircea.murar@muri.utcluj.ro (M.M.); bogdan.mocan@muri.utcluj.ro (B.M.); 2 Department of Internal Medicine, Iuliu Hațieganu University of Medicine and Pharmacy, 400012 Cluj-Napoca, Romania; mihaela.mocan@umfcluj.ro; 3 Department of Surgery, Iuliu Hațieganu University of Medicine and Pharmacy, 400012 Cluj-Napoca, Romania; vasile.bintintan@umfcluj.ro

**Keywords:** colorectal neoplasms, colorectal cancer surgery, intraoperative localisation, preoperative marking, radiofrequency identification, near-infrared fluorescence, indocyanine green, electromagnetic navigation, magnetic seed localisation, minimally invasive surgery, scoping review

## Abstract

**Background**: Precise intraoperative localisation of small colorectal tumours during laparoscopic surgery remains challenging due to absent tactile feedback and subserosal tumour location. Current standard methods, particularly India ink tattooing, demonstrate 15–30% failure rates for lesions less than 10 mm, leading to prolonged operative times, incomplete resections, and re-operations. Multiple emerging technologies promise improved localisation, yet comparative evidence remains fragmented. **Objective**: To map and characterise the current landscape of intraoperative marking and identification technologies for small colorectal tumour localisation during laparoscopic surgery, with emphasis on radiofrequency-based methods and alternative approaches, and to identify evidence gaps guiding future research. **Methods**: Following PRISMA-ScR guidelines, we systematically searched PubMed, Web of Science, and Scopus databases from January 2000 through December 2025 for studies evaluating tumour localisation technologies in colorectal cancer surgery, including primary tumour localisation during laparoscopic colectomy and localisation of colorectal liver metastases during hepatic surgery, or transferable anatomical applications with documented translational potential to colorectal surgery. Two independent reviewers screened all records, with discrepancies resolved through discussion and a third senior reviewer consulted for unresolved disagreements; data were extracted on technical performance, safety, feasibility, cost-effectiveness, usability, innovation potential, and evidence quality. **Results**: We included 89 studies comprising 18 colorectal-specific articles and 71 transferable/GI-adjacent studies. Detection success rates ranged from 71% to 100% across modalities. Near-infrared fluorescence with indocyanine green demonstrated the strongest clinical evidence with 75–100% detection across eight colorectal studies encompassing 2134 procedures and seamless workflow integration. Radiofrequency identification systems achieved 91.9–99% detection in feasibility studies with promising tissue penetration of 15–35 mm but limited colorectal validation. Electromagnetic navigation excelled in rigid organs with 85–98% success but showed degraded performance in mobile bowel at 71–75%. Critical evidence gaps included absent head-to-head comparative trials, non-standardised outcome metrics limiting cross-study comparability, and limited long-term safety data with only 14 studies providing follow-up exceeding six months. **Conclusions**: ICG fluorescence represents the most clinically mature technology identified, representing a priority candidate for colorectal-specific validation in challenging localisation scenarios. RFID systems demonstrate promising characteristics justifying prioritised research investment through adequately powered comparative trials. Future research must emphasise consortium-based comparative effectiveness studies, standardised outcome metrics, and integration with robotic and AI-assisted surgical platforms to accelerate clinical translation.

## 1. Introduction

Colorectal cancer remains a global health challenge, with increasing detection of small tumours through widespread screening programs. Early-stage colorectal cancers, neuroendocrine tumours, and complex polyps frequently require surgical resection, with minimally invasive laparoscopic approaches becoming the standard of care. However, the precise intraoperative localisation of these small lesions presents a significant technical challenge that directly impacts surgical outcomes. The absence of tactile feedback in laparoscopic surgery, combined with the small size and subserosal location of many colorectal tumours, creates a critical gap between endoscopic tumour identification and surgical resection.

The clinical consequences of inadequate intraoperative localisation are substantial and multifaceted. Studies of laparoscopic colorectal surgery demonstrate that difficulty in identifying small tumours leads to prolonged operative time, unplanned conversion to open surgery, incomplete resections with positive margins, and unnecessary extensive resections that compromise bowel function [1,2]. These complications increase healthcare costs, patient morbidity, and the need for re-operations. Current clinical practice relies heavily on preoperative imaging and endoscopic tattooing with India ink, yet these methods have well-documented limitations including dye migration, ambiguous localisation, and poor visibility through the serosal surface during laparoscopic approaches [1,3].

The fundamental challenge lies in the disconnect between endoscopic visualisation and surgical access. During colonoscopy, tumours are clearly visible from the intraluminal perspective, allowing precise marking of their location. However, during subsequent laparoscopic surgery performed from the extraluminal serosal surface, surgeons must rely on indirect indicators—anatomical landmarks, palpation (when possible), and previous marking—to locate the same lesion. This gap becomes particularly problematic for small tumours (<2 cm), deeply invasive lesions, and those located in anatomically complex regions such as the sigmoid colon or rectosigmoid junction. The need for precise, real-time, operator-independent localisation methods has driven innovation across multiple technological domains.

Recent years have witnessed the development of diverse technological approaches to address this localisation challenge. Optical methods, particularly near-infrared fluorescence imaging with indocyanine green (ICG), have gained widespread adoption in liver metastasis surgery and show promise for colorectal applications [2,4]. ICG enables real-time fluorescence visualisation during laparoscopy, though tissue penetration depth remains limited to approximately 8–10 mm from the surface [2,5]. Radiological approaches using metallic markers—including microcoils, radioactive seeds, and fiducial markers—offer excellent visibility on intraoperative fluoroscopy or computed tomography, but require coordination between radiology and surgery departments and may carry risks of marker migration [6,7,8]. Electromagnetic tracking systems represent an emerging paradigm, with radiofrequency identification (RFID) tags and magnetic markers enabling wireless detection through tissue without radiation exposure [7,9,10,11,12]. These technologies have evolved from simple passive marking systems to sophisticated active identification platforms capable of providing real-time spatial feedback to surgeons.

Despite these technological advances, gaps persist in the literature and clinical practice. First, the technology landscape remains fragmented, with no comprehensive synthesis comparing different approaches using standardised performance metrics. Studies typically focus on single technologies within specific clinical contexts, making cross-technology comparisons difficult. Second, most systems address either the marking phase or the detection phase, but few publications describe fully integrated workflows where endoscopists place markers during diagnostic colonoscopy, followed by laparoscopic detection from the serosal surface using dedicated devices [9,10,13]. Third, clinical translation data remains limited, particularly regarding detection performance through varying abdominal wall thicknesses, long-term marker safety profiles, standardised placement protocols, and cost-effectiveness analyses essential for healthcare system adoption [7,12].

The potential of radiofrequency identification technology deserves particular attention. RFID systems offer several theoretical advantages: electromagnetic waves penetrate tissue effectively, modern RFID tags can be miniaturised to sizes suitable for endoscopic deployment, and the technology is inherently resistant to electromagnetic interference in the operating room environment [9,12,14]. However, fundamental questions remain unanswered regarding optimal frequency ranges for tissue penetration versus tag size, antenna designs for omnidirectional detection, biocompatibility of miniature implantable tags, and clinically validated detection protocols for laparoscopic applications. Studies in breast cancer surgery have demonstrated RFID feasibility [7,12], but colorectal applications face unique challenges including bowel motility, pneumoperitoneum effects on tissue geometry, and the need for colonoscopic rather than percutaneous marker placement [9,10].

The primary objective of this scoping review is to comprehensively evaluate, synthesise, and critically appraise the current state-of-the-art in intraoperative marking and identification technologies for small colorectal tumour localisation during laparoscopic surgery. We employ a seven-domain evaluation framework encompassing technical performance, safety profile, clinical feasibility, cost-effectiveness, usability, innovation potential, and evidence quality, enabling granular comparison across heterogeneous technologies. This review explicitly emphasises the endoscopic-marking-to-laparoscopic-detection workflow, addressing the critical procedural gap between diagnosis and surgery. We provide dedicated analysis of radiofrequency identification methods, including technical physics considerations and biocompatibility requirements for endoscopic implantation, while also examining alternative approaches including optical fluorescence [2,5], electromagnetic navigation [15,16,17], and hybrid multimodal systems [18,19].

This synthesis aims to bridge the translational divide between engineering proof-of-concept studies and clinical implementation requirements, providing actionable insights for technology developers, colorectal surgeons, and healthcare administrators. By systematically mapping the current technology landscape, identifying implementation barriers, and proposing evidence-based directions for future research, this review aims to accelerate the development and clinical adoption of effective tumour localisation systems that can ultimately improve surgical precision, reduce complications, and enhance patient outcomes in minimally invasive colorectal surgery.

## 2. Methods

### 2.1. Protocol

This scoping review was registered with PROSPERO (CRD420261339085) and is reported following PRISMA-ScR guidelines. The protocol was amended during conduct to broaden the original scope—initially centred on radiofrequency identification technologies—to encompass all intraoperative marking and identification modalities for colorectal tumour localisation, reflecting the scarcity of colorectal-specific evidence for any single technology. To support the seven-domain narrative synthesis described in Section 2.6 and Section 2.7, risk of bias appraisal (Cochrane RoB 2, ROBINS-I) and GRADE-based evidence maturity characterisation were applied to key included studies, extending beyond standard scoping review methodology to provide additional analytical depth for clinical decision-making. The completed PRISMA-ScR checklist is provided as Supplementary File S3.

### 2.2. Eligibility Criteria

Studies were included if they evaluated any intraoperative marking or identification technology for tumour localisation in patients with colorectal cancer requiring surgical resection. Colorectal cancer surgery was defined broadly to encompass both laparoscopic colectomy for primary colorectal tumours (polyps, early-stage cancers, neuroendocrine tumours) and hepatic surgery for colorectal liver metastases, reflecting the full surgical pathway of colorectal cancer management. Additionally, studies examining similar localisation technologies in other anatomical sites (pulmonary, breast, hepatic) were included if the technology demonstrated clear transferability potential to colorectal applications based on comparable tissue characteristics, surgical approach, or detection principles. Eligible interventions included radiofrequency identification, electromagnetic navigation, optical fluorescence, physical markers, and hybrid systems placed either endoscopically or surgically. Comparators could include standard of care approaches such as preoperative imaging or India ink tattooing, other localisation methods, or no comparator in single-arm studies. Studies were required to report at least one quantitative or qualitative metric related to detection performance, safety, clinical feasibility, cost-effectiveness, usability, innovation potential, or evidence quality.

We included clinical studies of any design including randomised controlled trials, cohort studies, and case series with at least one patient, as well as preclinical studies with clear human translation pathways, systematic reviews, and technical reports with clinical validation data. Only English language publications from January 2000 to December 2025 were included to capture modern laparoscopic and endoscopic techniques. Studies were excluded if they addressed non-colorectal tumours without methodology transferable to colorectal applications, were pure ex vivo studies without described clinical translation pathways, were conference abstracts without full-text availability, or were duplicate publications, in which case the most complete version was retained.

### 2.3. Information Sources and Search Strategy

We systematically searched three major databases from inception through December 2025: PubMed (MEDLINE), Web of Science (Core Collection), and Scopus. The search strategy combined free-text terms across four domains: anatomical terms including colorectal, colon, rectal, rectum, bowel, and intestinal; pathological terms including tumour, neoplasm, polyp, lesion, cancer, adenoma, and carcinoma; procedural terms including laparoscopy, laparoscopic surgery, minimally invasive surgery, endoscopy, colonoscopy, and intraoperative; and technological terms including localisation, identification, marking, tattoo, RFID, radiofrequency, electromagnetic, magnetic, fluorescence, ICG, indocyanine green, microcoil, seed, clip, marker, tracer, navigation, and tracking. Complete search strategies for all three databases are provided in Appendix A.

### 2.4. Study Selection Process

Two independent reviewers screened all titles and abstracts using predefined eligibility criteria. Articles meeting inclusion criteria or with uncertain eligibility advanced to full-text review, conducted independently by both reviewers. Discrepancies were resolved through discussion, with a third senior reviewer consulted for unresolved disagreements. The selection process was documented in a PRISMA flow diagram.

### 2.5. Data Extraction and Synthesis

A standardised data extraction form was developed a priori and piloted on five representative studies before full implementation. One reviewer extracted data with verification by a second reviewer.

Extracted data elements included study characteristics such as publication year, country, study design, sample size, and patient demographics; tumour characteristics including size, location (cecum, ascending, transverse, descending, sigmoid, rectum), histology, and stage; and technology characteristics encompassing marking technique (endoscopic versus intraoperative), marker type and specifications, detection device and method, and reported challenges. Outcome metrics were extracted across seven evaluation domains. Technical performance (Category 1) included detection rate, localisation precision, sensitivity/specificity, detection range, and tissue penetration depth. Safety profile (Category 2) encompassed complication rates, adverse reactions, marker migration, and biocompatibility. Clinical feasibility (Category 3) covered technical complexity, time requirements, equipment needs, and workflow integration. Cost-effectiveness (Category 4) included per-patient costs, infrastructure investment, and operative time impact. Usability (Category 5) addressed operator interface quality, learning curves, and inter-operator consistency. Innovation potential (Category 6) evaluated technological novelty, scalability, and integration with emerging platforms. Evidence quality (Category 7) assessed study design rigour, sample size adequacy, follow-up duration, and comparative design.

### 2.6. Study Characterization and Methodological Quality Appraisal

Given the heterogeneous nature of included studies spanning clinical trials, case series, and technical reports across diverse technologies and anatomical applications, we characterised methodological quality through expert assessment by clinical co-authors with subspecialty expertise in laparoscopic colorectal surgery. Key features documented for each study included design type, sample size, presence of comparative groups, outcome measurement approaches, follow-up duration, potential conflicts of interest including industry funding, and single-institution versus multicenter conduct.

To assess the relevance of each included study to the review’s core objective, a structured inclusion justification score (scale 1–10) was assigned to each study by two reviewers, evaluating whether the study informed at least one of the seven predefined evaluation domains in the context of colorectal laparoscopic tumour localisation. Full scoring criteria and justification arguments for all included studies are provided in Supplementary Table S1 (complete data extraction matrix, column 0.C). Studies from adjacent anatomical domains were additionally assessed for transferability potential based on tissue characteristics, surgical approach similarity, and detection principle compatibility. This scoring informed the narrative synthesis weighting and supported proactive exclusion of studies with insufficient domain coverage prior to manuscript finalisation.

The seven-domain evaluation framework employed in this review was developed a priori drawing on established technology assessment methodologies for surgical innovation. The framework structure aligns with the IDEAL framework for surgical innovation [20], which emphasises staged evaluation of device feasibility, safety, efficacy, and effectiveness. Domains 1–2 (technical performance and safety) correspond to IDEAL stages 1–2a assessment criteria. Domains 3–5 (feasibility, cost-effectiveness, usability) reflect health technology assessment dimensions recommended by MAUDE and HTAi core models. Domain 6 (innovation potential) draws on IDEAL-D criteria for device-specific innovation readiness. Domain 7 (evidence quality) applies established Oxford CEBM evidence hierarchy principles. This multi-domain approach is consistent with published systematic reviews of surgical localisation technologies [5,21].

Methodological quality of the randomised controlled trials was appraised using the Cochrane RoB 2 tool across five domains: randomisation process, deviations from intended interventions, missing outcome data, measurement of the outcome, and selection of the reported result. Methodological quality for a pre-specified subset of non-randomised studies was appraised using the ROBINS-I tool across seven domains; this subset comprised all directly applicable colorectal clinical studies with patient-level outcome data and the largest clinical cohort studies for the three most clinically mature emerging transferable technologies—RFID localisation and combined magnetic localisation. For preclinical, ex vivo, and technical feasibility studies where standard risk of bias tools are not applicable, the structured relevance and quality scoring framework described above was applied.

Evidence maturity for main technology outcomes was characterised using the GRADE approach. GRADE-based evidence maturity characterisation was performed for the primary detection outcome of technology groups with sufficient clinical evidence for synthesis, defined as those represented by at least two clinical studies with extractable detection performance data. Downgrade and upgrade decisions followed published GRADE guidance across five domains: risk of bias, inconsistency, indirectness, imprecision, and publication bias.

### 2.7. Data Synthesis Strategy

We employed structured narrative synthesis as the primary analytical approach. This approach reflects three documented sources of heterogeneity in the included evidence: clinical heterogeneity (diverse anatomical sites, surgical approaches, and patient populations), methodological heterogeneity (RCTs, prospective single-arm trials, retrospective cohorts, and preclinical studies), and outcome heterogeneity (detection rate definitions encompassing intraoperative marker retrieval, complete surgical resection, lesion localisation success, and treatment technical success—as detailed in Section 3.3.2).

Our synthesis framework organised findings across seven predefined evaluation categories, each encompassing multiple specific metrics extracted systematically from included studies. Category 0 (General Aspects) captured intraoperative marking techniques, marker types, surgical tracking technologies, and reported challenges. Category 1 (Technical Performance) included eight metrics: detection rate, localisation precision, sensitivity/specificity, spatial resolution, detection range, tissue penetration, interference susceptibility, and device sterilisation. Category 2 (Safety Profile) encompassed six safety domains: complication rates, adverse reactions, long-term effects, material biocompatibility, marker size and migration risk, and retention duration. Category 3 (Clinical Feasibility) evaluated six workflow dimensions: technical complexity, time requirements, equipment needs, compatibility with existing systems, marking-to-surgery time intervals, and personnel requirements. Category 4 (Cost-Effectiveness) analysed six economic factors: per-patient costs, infrastructure investment, indirect costs, operative time savings, complication reduction, and reoperation avoidance. Category 5 (Usability) assessed five human factors: operator interface quality, real-time feedback, ergonomics, inter-operator consistency, and procedure standardisation. Category 6 (Innovation Potential) examined five translational aspects: approach uniqueness, scalability, compatibility with emerging technologies, technical barriers, and adoption barriers. Category 7 (Evidence Quality) characterised five study features: design level, sample size, follow-up duration, comparative methodology, and metric standardisation.

Data were synthesised narratively within each category, with cross-category integration to provide comprehensive technology assessments. Studies were weighted narratively according to their inclusion justification score (Supplementary Table S1, column 0.C), study design hierarchy, and ROBINS-I or RoB 2 risk of bias judgment, with directly applicable colorectal studies given primary interpretive weight over transferable evidence. Where detection rates are compared across technologies, these reflect heterogeneous endpoints in distinct clinical contexts rather than equivalent comparative metrics, as noted in Section 3.3.2. Given the preponderance of early-phase feasibility studies and single-arm designs, we emphasised description of performance ranges, identification of knowledge gaps, and assessment of clinical translation readiness rather than definitive comparative effectiveness conclusions.

Evidence maturity for technology groups with sufficient clinical data for synthesis was characterised using the GRADE approach, with results presented in Section 3.3.8. A technology-level evidence synthesis summarising findings across all nine technology groups and seven evaluation domains is provided in Supplementary Table S2.

## 3. Results

### 3.1. Study Selection and Inclusion

**Figure 1:**
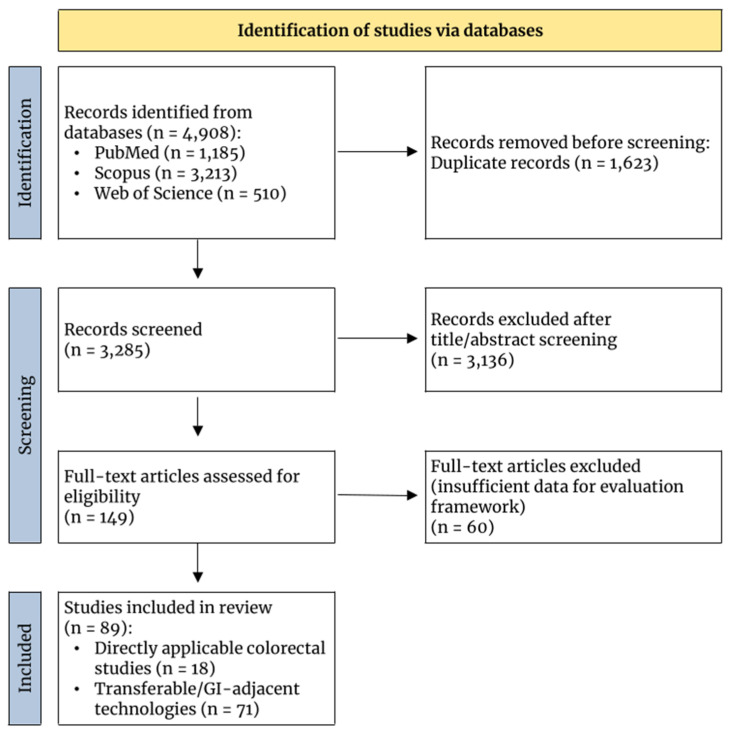
The PRISMA flow diagram for this scoping review.

The systematic search identified 4908 records across three electronic databases (PubMed—1185 records; Web of Science—510 records; Scopus—3213 records), which underwent title and abstract screening, resulting in 149 full-text articles assessed for eligibility. Of these, 89 studies met the inclusion criteria and underwent full data extraction using our structured seven-category framework. The 60 studies excluded from final synthesis lacked sufficient data granularity for our seven-category evaluation framework. The PRISMA flow diagram documenting the complete selection process is presented in Figure 1. No additional studies were identified through citation searching of included articles or manual searches of conference proceedings. Characteristics of all 89 included studies, including study design, sample size, technology type, and application, are presented in Table 1 (see end of manuscript, before References).

The remaining 89 studies were further categorised based on their direct applicability to laparoscopic colorectal tumour detection. Eighteen studies were classified as directly applicable, focusing specifically on radiofrequency, electromagnetic, or alternative identification methods for colorectal lesions during laparoscopic surgery [1,2,6,9,10,15,17,18,19,22,23,24,25,26,27,28,29,30]. The remaining 71 studies were categorised as transferable or gastrointestinal-adjacent applications, encompassing techniques and technologies from related surgical domains that demonstrate potential for adaptation to colorectal tumour localisation. All included studies underwent structured data extraction according to predefined evaluation criteria spanning general aspects, detection performance, safety outcomes, procedural complexity, economic considerations, user experience, innovation potential, and evidence quality.

### 3.2. Study Characteristics

#### 3.2.1. Directly Applicable Studies

Among the 18 directly applicable studies, technological approaches were diverse. Navigation systems integrating electromagnetic tracking with preoperative imaging dominated the literature, with studies employing electromagnetic patches [15], optical stereotactic navigation with deep learning-assisted segmentation [17], and 3D image-guidance systems using tracked ultrasound [18,24]. Near-infrared fluorescence imaging emerged as a prominent optical technique, utilising indocyanine green administered 1–2 days preoperatively [2], targeted ICG-loaded nanoparticles [27], and FDA-approved fluorescent dyes including Cytalux and Gleolan [29]. Radioguided surgery was represented through gamma probe-based approaches with hybrid tracers combining radioactive and fluorescent properties [19].

Electromagnetic and inductive proximity sensor technologies constituted a distinct category. Novel inductive sensor platforms optimised for laparoscopic detection of modified hemostatic clips demonstrated feasibility in preclinical and ex vivo human specimens [9,10]. Traditional marking approaches included preoperative colonoscopic tattooing with India ink or metallic clip placement [1], and CT-guided placement of straightened McKenzie-Diener silver clips [6]. Thermal ablation studies, while focused on treatment rather than detection per se, employed ultrasound guidance for precise tumour localisation during radiofrequency and microwave ablation [22]. US/MRI fusion guidance utilising pre-chemotherapy imaging as a reference represented marker-less navigation strategies [30].

The marker types employed varied substantially. Physical metallic markers included standard and titanium hemostatic clips [9], modified clips with nanometric metallic coatings [10], and silver metal clips [6]. Optical fluorophores ranged from non-targeted ICG to peptide-functionalised nanoparticle systems [27] and receptor-targeted probes [29]. Radioactive tracers comprised 99mTc-nanocolloid, 125I-labeled seeds, and hybrid ICG-[99mTc]Tc-albumin formulations [19]. Several approaches avoided physical markers entirely, relying instead on virtual 3D models created from preoperative imaging [17,24] or previously visible metastases as virtual targets [18].

#### 3.2.2. Transferable/Adjacent Studies

The 71 transferable and GI-adjacent studies predominantly derived from pulmonary nodule localisation (n = 22), breast lesion localisation (n = 12), hepatic tumour localisation (n = 10), and general intraoperative navigation technologies applicable across multiple anatomical sites (n = 6). Pulmonary applications demonstrated advanced radiofrequency identification systems, with RFID tags placed under imaging guidance and detected intraoperatively using dedicated probes [7,12]. Electromagnetic tracking systems employed in thoracic surgery included tracked fiducials with embedded EM position sensors [31] and magnetic seed technologies combined with superparamagnetic tracers [11].

Breast surgery literature contributed sophisticated wireless localisation platforms, including radar-based systems and electromagnetic chip technologies enabling bracket-based localisation of margins [32]. Injectable optical tracers received extensive investigation in both pulmonary and breast applications, with navigational bronchoscopy-guided ICG:albumin injection creating fluorescent tattoos [33], and receptor-targeted near-infrared probes under phase II/III clinical evaluation [34].

Software-based deformable image registration techniques, validated in hepatic thermal ablation procedures, demonstrated biomechanical modelling approaches for compensating tissue deformation [35].

Wire-based localisation remained prevalent across multiple GI-adjacent domains, employing CT-guided placement of microcoils, hook-wires, and spiral-wire configurations [5,34,36]. Hybrid marker systems combined modalities, such as simultaneous use of magnetic seeds and superparamagnetic lymphatic tracers [11], or dual radioactive-fluorescent tracers for sentinel node mapping [19]. Emerging technologies included augmented reality navigation platforms, artificial intelligence-assisted segmentation for surgical planning, and integration with robotic surgical systems [5,36].

### 3.3. Classification by Evaluation Criteria

#### 3.3.1. General Aspects

**Figure 2:**
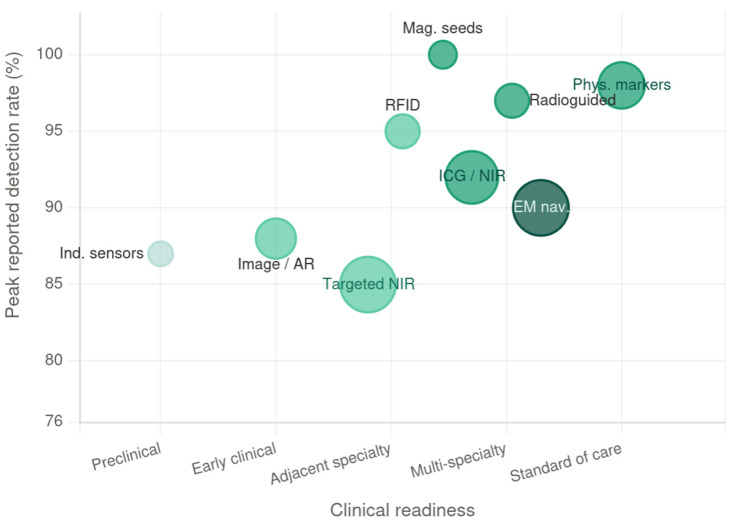
Technology evidence map for intraoperative tumour localisation systems evaluated in this review. Bubble position indicates clinical readiness (x-axis) and peak reported detection rate (y-axis). Bubble area is proportional to the number of supporting studies assigned to each technology group (Supplementary Table S2). Shading represents evidence quality tier: darkest = phase-3 RCT evidence in colorectal surgery; medium-dark = clinical evidence comprising RCT in adjacent specialty or multiple colorectal clinical series; medium-light = predominantly observational or early-phase clinical data; lightest = preclinical and ex-vivo evidence only. The two NIR fluorescence groups (non-targeted ICG, n = 10; targeted probes, n = 11) collectively represent the largest technology portfolio in the corpus (n = 21 with possible overlap per Supplementary Table S2 allocation note). Detection rates represent midpoints of documented performance ranges; positions within clinical readiness categories are approximate. AR, augmented reality; EM nav., electromagnetic navigation; ICG, indocyanine green; NIR, near-infrared; RFID, radiofrequency identification.

Synthesised findings aggregated by technology type across all seven domains are available in Supplementary Table S2. An integrated visual summary of technology group positioning by clinical readiness and peak detection performance is presented in Figure 2.

*Directly Applicable Studies*

Marking and identification techniques among directly applicable studies encompassed four principal modalities. Electromagnetic navigation systems constituted the largest category, utilising either external tracking patches temporarily attached to the patient for registration [15], or permanent virtual models derived from preoperative imaging without physical markers [17,18,24,30]. Optical imaging approaches predominantly employed near-infrared fluorescence with ICG-based agents [2,27,29], supplemented by autofluorescence and cathepsin-activatable fluorescent probes [26]. Radioguided surgery methodologies incorporated gamma probes and portable gamma cameras, with some protocols employing freehand SPECT capabilities [19]. Physical marker placement via endoscopic or percutaneous routes remained relevant, utilising metallic clips [1,6,9,10] or injectable dyes [1].

Surgical tracking and navigation technologies demonstrated substantial heterogeneity. Electromagnetic tracking systems employed NDI Aurora platforms with 5-degree-of-freedom position sensing [15], while optical tracking utilised infrared cameras for stereotactic navigation [17]. Ultrasound-based navigation incorporated real-time fusion with preoperative CT/MRI [18,24], and some systems integrated deep learning algorithms for automated 3D segmentation [17]. Thermal ablation studies relied on conventional ultrasound visualisation without dedicated navigation [22]. Advanced imaging fusion combined anatomical landmarks from pre-chemotherapy MRI with intraoperative ultrasound findings [30].

Reported challenges varied by technological approach. Navigation systems faced difficulties with tissue deformation between preoperative imaging and surgery, requiring continuous registration updates [15,17]. Disappearing colorectal liver metastases following chemotherapy presented substantial localisation challenges, with 16% successfully identified using image guidance [18] and systematic fiducial marker placement recommended for high-risk lesions [28]. Inductive proximity sensors required optimisation of electromagnetic probe design for adequate tissue penetration and resolution [9,10]. Near-infrared fluorescence imaging demonstrated variable tumour-to-background ratios dependent on probe accumulation kinetics [2,27], while radioguided approaches encountered limitations in spatial resolution for closely-spaced lesions [19]. Colonoscopic tattooing exhibited marker migration and unpredictable spreading patterns [1].

*Transferable/Adjacent Studies*

Transferable technologies from pulmonary and breast applications reported similar challenges. RFID systems faced depth-dependent signal attenuation requiring precise probe positioning [7,12]. Magnetic seed localisation reported depth limitations for deeply seated tumors (>3.5 cm) and potential seed migration concerns [11]. Wire-based techniques risked dislodgement, pneumothorax in pulmonary applications, and inadequate margin assessment [5,32,34]. Electromagnetic tracking systems faced challenges with significant tissue deformation between imaging and operative states, particularly in deflated and manipulated lung tissue [31]. Navigational bronchoscopy for marker placement demanded advanced technical skills and coordination between pulmonology and surgical teams [33]. Deformable image registration algorithms addressed challenges of hepatic motion and visual assessment variability [35].

#### 3.3.2. Detection Performance

Detection rate definitions varied substantially across studies, encompassing percentage of markers successfully identified intraoperatively, complete surgical resection achievement, lesion localisation success, and treatment technical success, thereby precluding direct cross-technology quantitative comparison. The following figures are reported as descriptive ranges within each technological context rather than as equivalent comparative metrics.

*Directly Applicable Studies*

Electromagnetic navigation achieved 85% complete lymph node removal in targeted retroperitoneal dissection [15], while image-guided localisation identified 16% of sonographically occult disappearing liver metastases [18]. Inductive proximity sensors demonstrated 85% detection rates for 0.9 mm stainless steel clips in wet-laboratory conditions, 90% in porcine models, and 85% in fresh human surgical specimens [9]. Modified hemostatic clips with nanometric coatings achieved 80% detection in 30 cm bowel segments and 75–90% in human gastric specimens dependent on operator experience [10]. Thermal ablation modalities reported successful treatment in 92.9% of radiofrequency and 92.7% of microwave ablation lesions [22]. Near-infrared fluorescence imaging with ICG demonstrated high detection rates, with 83% overall sensitivity for colorectal liver metastases and 100% detection for superficial lesions (<8 mm subcapsular depth) [2], while NIR-II imaging achieved 100% detection for hepatic tumours [29].

Localisation precision metrics, when reported, indicated millimeter-scale accuracy for several modalities. Electromagnetic navigation systems achieved near-complete lymph node removal with 93% probability of successful removal per target node [15]. Image-guided systems demonstrated millimeter resolution during needle transit and accurate segment-specific targeting [18]. Inductive proximity sensors provided millimeter precision when the probe contacted the serosa, requiring scanning within a 15 cm search area for reliable detection [9], with ~5 mm discrimination distance between separate detection points [10]. Ultrasound-guided thermal ablation precision depended on operator visualisation capabilities rather than quantified measurement [22].

Sensitivity and specificity data for associated imaging methods were selectively reported. Image-guidance systems employing tracked ultrasound with optical navigation demonstrated capability to locate radiographically identified but sonographically occult lesions [18,24], though formal diagnostic accuracy metrics were not provided. Near-infrared fluorescence approaches varied in diagnostic performance based on targeting strategies, with receptor-targeted probes (Cytalux for folate receptor-alpha) showing enhanced tumour-specific uptake compared to non-targeted ICG [29]. A review of radioguided surgery innovations reported sentinel lymph node detection sensitivities exceeding 95% in established protocols, though colorectal-specific data were limited [19].

Detection range among directly applicable technologies varied substantially by modality. Electromagnetic tracking maintained accuracy within a 20–40 cm effective range covering the retroperitoneal and pelvic surgical field [15], while inductive proximity sensors required probe positioning within 15 cm of targets for optimal signal detection [9]. Radioguided techniques using gamma probes detected signal at ranges of several centimetres depending on radiotracer dose and tissue depth [19].

Tissue penetration characteristics influenced detection reliability in directly applicable studies. Near-infrared fluorescence at 785–820 nm wavelength penetrated 8–10 mm of tissue [2,27,29], while NIR-II wavelengths (900–1800 nm) offered superior depth penetration with reduced scattering [29]. Inductive sensor performance degraded with fat interposition, failing with fat layers exceeding 2 mm and requiring probe-serosa contact for detection [9].

Interference susceptibility varied by technology platform. Electromagnetic navigation systems demonstrated interference from metallic implants and external electromagnetic fields, requiring patient exclusion criteria and careful operating room setup [15], with one infrared camera malfunction reported during patient repositioning [17]. Optical tracking required unobstructed line-of-sight, with surgical instruments potentially blocking camera views [18,24]. Near-infrared fluorescence systems showed minimal interference with standard surgical equipment [2,29], though blood pooling, cauterised tissue, and tissue autofluorescence degraded signal quality [26,29].

Sterilisation protocols among directly applicable technologies generally accommodated existing workflows, with optically tracked instruments and gamma probes designed for standard surgical sterilisation and reuse [19,24]. ICG required sterile preparation for intravenous injection, with camera systems utilising sterile draping for intraoperative deployment [2]. Nanoparticle formulations were prepared under sterile conditions without specialised sterilisation requirements beyond conventional protocols [27].

*Transferable/Adjacent Studies*

Transferable technologies from pulmonary nodule and breast lesion localisation provided extensive quantitative performance data. RFID tag systems achieved 99.0% successful intraoperative detection and retrieval [12], though comparative studies showed 91.9% success rates with some tags requiring subsequent wire placement [7]. Magnetic seed detection rates reached 100% in appropriately positioned lesions [11]. Wire-based localisation demonstrated variable success rates, with microcoil retrieval at 95–98% and hook-wire at 91–96%, though complications included wire migration and transection [32,34]. Radar-based wireless localisation achieved 97.7% retrieval rates with lower migration risk compared to wire techniques [32].

Detection range and tissue penetration data from adjacent applications extended the performance picture. Electromagnetic tracking systems enabled localisation throughout the thoracic cavity, successfully tracking nodules at depths ranging from superficial to 40 mm [31]. RFID systems demonstrated 30 mm maximum communication range between marker and detection probe [37], with adequate penetration confirmed through varying breast tissue densities across clinical series [7,12]. Magnetic seed detectors functioned effectively at depths up to 3.5 cm [11]. Radioguided techniques using gamma probes detected signal at ranges up to several centimetres depending on radioactivity dose [34].

RFID tags produced MRI artifacts complicating neoadjuvant therapy monitoring in patients receiving systemic treatment [7], while magnetic seeds were contraindicated in patients carrying pacemakers or defibrillators [34]. Regarding sterilisation, RFID tags and wire localisation devices employed pre-sterilised single-use configurations [7,12].

#### 3.3.3. Safety and Biocompatibility

*Directly Applicable Studies*

Complication rates among directly applicable studies were generally low for established techniques but varied with invasiveness of marker placement. Electromagnetic navigation systems using external patches reported no marker-related complications and demonstrated safety profiles comparable to conventional surgery [15]. Image-guided ultrasound navigation demonstrated no adverse events attributable to the optical tracking system itself [18,24]. Inductive sensor studies in preclinical and ex-vivo models reported no tissue injury from electromagnetic field exposure [9,10]. Thermal ablation series documented major complications (grade ≥ 3) in 4.9% of radiofrequency ablation and 7.6% of microwave ablation patients, primarily comprising liver-related events including bilomas and abscesses [22].

Colonoscopic tattooing complications were not quantified in directly applicable colorectal studies, though the procedure was generally well-tolerated [1]. CT-guided clip placement for hepatic metastases resulted in minor pneumothorax in one of three patients (33%), which resolved spontaneously without intervention [6]. Near-infrared fluorescence imaging using ICG demonstrated excellent safety, with adverse reactions occurring in fewer than 1 in 40,000 patients and no ICG-related adverse events reported in colorectal liver metastasis cohorts [2]. ICG allergic reactions were described as extremely rare, with the agent showing good safety profiles across multiple applications [29]. Radioguided surgery demonstrated established safety profiles with low morbidity [19].

Adverse reactions to marker materials were infrequent. Metallic clips composed of titanium, stainless steel, or silver with biocompatible coatings demonstrated established safety profiles without adverse reactions in short-term assessment [6,9,10]. ICG exhibited safety across diverse formulations, including nanoparticle-encapsulated preparations with FDA-approved biodegradable PLGA-PEG carriers [27]. Peptide-functionalised targeting moieties underwent toxicity screening without acute adverse effects in preclinical models, maintaining cell viability >83% and showing no tissue morphological damage [27]. Receptor-targeted optical probes (Cytalux, Gleolan) demonstrated good safety profiles and were well-tolerated in clinical evaluations [29].

Long-term effects and marker migration received limited documentation in directly applicable colorectal studies. India ink tattoos provided permanent or months-long visibility, though long-term marker effects were not systematically tracked [1]. Metallic clips remained permanently in liver tissue with stable radiographic visibility and theoretical low risk of dislocation in solid organs [6]. Optical fluorophores exhibited clearance kinetics dependent on formulation, with ICG clearing within days [2] while nanoparticle preparations remained detectable up to 72 h post-injection [27]. Radioactive seeds remained in tissue long-term with minimal radiation exposure and no requirement for retrieval [19].

*Transferable/Adjacent Studies*

Safety data from pulmonary and breast applications provided additional perspective on marker-related risks. RFID tag placement demonstrated excellent safety, with no tag-related complications reported and post-operative complications (seromas, wound infections) in 1.7% of cases [12]. Magnetic seed localisation showed no seed migration, with one major complication unrelated to the seed itself [11]. Wire-based techniques exhibited pneumothorax rates of 4–32% and haemorrhage in 0.6–14.8% of percutaneous placements, with wire displacement requiring occasional conversion to thoracotomy [5,34]. Injectable dye techniques showed zero adverse events in navigational bronchoscopy applications [33], though rare anaphylaxis to methylene blue has been reported in literature [5].

#### 3.3.4. Procedural Complexity

*Directly Applicable Studies*

Technical complexity of marker placement varied substantially across modalities in directly applicable studies. Electromagnetic navigation systems required high-complexity setup involving CBCT scanning and electromagnetic calibration [15], with ongoing intraoperative registration adjustments [17]. Optical stereotactic navigation demonstrated median preparation time of 26 min for system setup and calibration [17]. Colonoscopic tattooing demanded experienced endoscopist expertise for precise circumferential dye injection [1]. CT-guided metallic clip placement necessitated interventional radiology expertise and image-guided percutaneous access [6]. Near-infrared fluorescence approaches using systemically administered ICG required minimal additional technical skill beyond agent injection and camera operation [2].

Time requirements demonstrated considerable range when quantified. Electromagnetic navigation setup added CBCT scanning and calibration time, though total operative time was not prolonged significantly [15]. Image-guidance systems required median registration time of 62 s [18] to 1.6 min [24], with ultrasound-based tumour identification adding median 4.5 min [24]. ICG administration occurred 1–2 days preoperatively, with no significant increase in operative time compared to standard surgery and rapid surface screening completed in less than 5 min [2]. Preoperative colonoscopic tattooing and CT-guided clip placement added time to preparatory procedures, though specific durations were not systematically reported [1,6].

Equipment capital requirements varied substantially. Near-infrared fluorescence systems ranged from USD 30,000 to USD 300,000 [2,29]. Electromagnetic navigation required dual-phase CT, intraoperative CBCT, field generators, tracked instruments, and navigation software [15]. Image-guidance systems cost between USD 150,000 and USD 200,000 [24]. Inductive proximity sensors remained in prototype development [9,10]. Radioguided surgery necessitated gamma probes, cameras, and radiation safety infrastructure [19].

Compatibility with existing laparoscopic equipment varied by technology. Near-infrared fluorescence cameras integrated seamlessly with standard laparoscopic and robotic platforms [2,26]. Inductive proximity sensors were fully compatible with existing trocars and clip appliers [9,10]. Image-guidance systems required additional optical tracking infrastructure but utilised standard ultrasound equipment [18,24]. Electromagnetic navigation demanded dedicated operating room space for CBCT and field generators [15].

*Transferable/Adjacent Studies*

Procedural complexity data from breast and thoracic applications consistently demonstrated that wireless localisation systems required lower placement complexity and offered greater scheduling flexibility than wire-guided techniques, with marker placement decoupled from the operative day and performable during routine clinical visits [7,11,12,38,39]. However, these complexity profiles reflect percutaneous or stereotactic placement in breast tissue and would change substantially when ported to colorectal applications where endoscopic submucosal deployment during colonoscopy constitutes the natural placement route. One system was specifically engineered for this context: the magnetically stabilised injectable hydrogel designed for hollow viscera employed endoscopic submucosal injection as the placement method, with a 5 mm diameter laparoscopic-compatible sensor probe for detection, offering a procedural complexity profile directly analogous to established colorectal endoscopic marking workflows [13]. Near-infrared fluorescent marking clips demonstrated compatibility with robotic da Vinci Firefly systems in gastric cancer surgery without modification to existing surgical setup [40], directly relevant to robotic colorectal platforms. The Sentimag magnetic detection probe was confirmed compatible with standard laparoscopic instrumentation in breast applications [11], providing indirect evidence of laparoscopic feasibility pending colorectal-specific validation.

#### 3.3.5. Economic Evaluation

*Directly Applicable Studies*

Direct cost-per-patient data remained incompletely reported, as summarised in Table 2 (see end of manuscript, before References). Electromagnetic navigation incurred considerable per-patient costs for CBCT scanning and tracking, though specific figures were not quantified [15]. Near-infrared fluorescence using ICG cost USD 100–300 per patient [2], while investigational targeted probes ranged from USD 1000 to USD 5000 [29]. Colonoscopic tattooing materials cost USD 20–50 for dye or USD 100–200 for clips [1]. Radioguided surgery added radiopharmaceutical costs of USD 100–1000, plus SPECT/CT imaging (USD 500–1500) [19].

Infrastructure investments varied substantially. Near-infrared fluorescence systems cost USD 30,000–300,000 depending on sophistication [2,29]. Electromagnetic navigation required significant investment for CT/CBCT integration, field generators, and workstations, though costs were not quantified [15]. Image-guidance systems with optical tracking cost USD 150,000–200,000 [24], while advanced navigation platforms with 3D C-arm and deep learning capabilities ranged from USD 500,000 to USD 900,000 [17].

Indirect costs included training, maintenance, and technical support. Electromagnetic navigation required surgeon training in system operation and ongoing software licensing and technical support [15]. Near-infrared fluorescence demonstrated minimal training needs (1–2 cases) [2]. Radioguided approaches required radiation safety training, quality control protocols, and interdepartmental coordination [19].

Potential cost savings received limited quantification. Electromagnetic navigation achieved 85–97% complete target removal, reducing repeat procedures [15], though setup time offset operative efficiency gains. Image-guidance potentially avoided futile laparotomies by enabling complete identification of disappearing metastases [18]. Despite these benefits, comprehensive cost-effectiveness modelling remained sparse, with most studies focusing on technical feasibility rather than formal economic evaluation.

*Transferable/Adjacent Studies*

Cost and economic data from breast and thoracic localisation literature consistently demonstrated a conceptual offset model in which higher per-unit costs of wireless technologies were compensated by reductions in repeat procedures, reoperations, and same-day scheduling overhead compared to wire-guided localisation [7,11,12,32,38]. The underlying economic logic is transferable to colorectal applications: more reliable intraoperative localisation reduces conversion to open surgery, incomplete resections, and reoperations, each of which carry substantially greater costs than the marker itself. However, the specific figures reported in breast and thoracic literature are not directly applicable to colorectal surgical economics given fundamental differences in procedure type, reimbursement structures, and failure mode costs. Formal cost-effectiveness modelling specific to colorectal tumour localisation, incorporating quality-adjusted outcomes and full pathway costs, is absent from the current literature and represents a priority research gap.

All cost figures are reported as cited in the original studies; given the review spans publications from 2007 to 2025, figures from earlier studies should be interpreted as indicative rather than current, as equipment and pharmaceutical costs may have changed substantially over this period.

#### 3.3.6. User Experience

*Directly Applicable Studies*

Operator interface characteristics varied across technological platforms in directly applicable studies. Electromagnetic navigation systems provided 3D anatomical visualisations with real-time instrument tracking and colour-coded anatomy, receiving high usability ratings from surgeons [15,17]. Image-guidance platforms combining ultrasound with optical tracking offered complex 3D visual overlays requiring interpretation skills and learning curves for spatial understanding of fused images [18,24]. Near-infrared fluorescence systems integrated seamlessly with existing laparoscopic visualisation, toggling between white light and fluorescence modes via foot pedal control with intuitive real-time colour overlay [2,29], though camera head size occasionally limited posterior liver access [2]. Inductive proximity sensors provided simple acoustic and visual feedback indicating probe-to-target distance with intuitive operation [9,10].

Real-time feedback quality varied across platforms. Electromagnetic navigation provided continuous instrument tracking with high surgeon confidence [15,17]. Near-infrared fluorescence offered instantaneous visual feedback upon lesion exposure with sustained signal throughout resection [2,27]. Radioguided techniques delivered continuous audible count-rate information, with advanced systems providing real-time 2D scintigraphic imaging and 3D navigation capabilities [19].

Procedure standardisation varied by technology maturity. Electromagnetic navigation and image-guidance systems demonstrated protocolised workflows with defined registration and guidance procedures [15,17,24]. Colonoscopic tattooing followed established guidelines despite surgeon discretion [1], and CT-guided clip placement employed reproducible protocols [6]. Near-infrared fluorescence lacked consensus standardisation, with variable ICG dosing and timing, though hepatic surgery guidelines emerged [26]. Investigational technologies including inductive sensors and targeted probes had experimental protocols awaiting clinical validation [9,10,23].

*Transferable/Adjacent Studies*

Device-level interface and ergonomic characteristics of wireless localisation systems represent the most anatomy-independent aspect of usability and therefore the most directly informative for colorectal adaptation assessment. RFID detection systems employed simple handheld readers providing continuous numeric distance readouts and auditory proximity feedback, with consistent detection performance confirmed across multiple operators [7,12]. In thoracic applications, the RFID detection probe measured 10 mm in diameter—compatible with standard laparoscopic trocar sizing—and provided auditory pitch-based feedback requiring no visual display integration, allowing surgeons to maintain operative field attention [37]. The magnetically stabilised injectable hydrogel detection system used a 5 mm electromagnetic probe specifically designed to match laparoscopic instrument dimensions, with PC-based 3D real-time visualisation, directly addressing laparoscopic ergonomic requirements [13]. Near-infrared fluorescent marking clips demonstrated seamless integration with the da Vinci Firefly console without additional devices or workflow interruption in robotic gastrointestinal surgery [40]. Magnetic seed detection via the Sentimag probe provided auditory signal intensity feedback that surgeons unanimously rated as easy to learn, with laparoscopic probe compatibility confirmed [11]. Procedure standardisation protocols and specialty-specific learning curve data from these applications reflect breast and thoracic surgical contexts and are not directly transferable to colorectal practice.

#### 3.3.7. Innovation and Adoption Potential

*Directly Applicable Studies*

Unique innovations among directly applicable technologies included several notable advances. Inductive proximity sensor platforms represented novel electromagnetic detection principles optimised for surgical environments, distinguishing themselves from conventional radiofrequency or magnetic approaches through frequency-selective resonance [9,10]. Peptide-functionalised ICG-loaded nanoparticles introduced targeted drug delivery concepts to intraoperative imaging, combining enhanced permeability and retention with active receptor binding [27]. Deep learning-assisted automated segmentation for surgical navigation offered workflow streamlining compared to manual segmentation requirements [17]. Hybrid radioactive-fluorescent tracers enabled dual-modality detection, combining gamma probe sensitivity with fluorescence-guided precision [19].

Scalability potential varied by technological maturity. Near-infrared fluorescence demonstrated broadest applicability, with FDA-approved dyes extending across multiple organ systems and surgical platforms [29]. Electromagnetic navigation supported extension to other high-precision resections [15]. Inductive proximity sensors showed scalability throughout the gastrointestinal tract with potential for other mucosal-accessible organs [9,10].

Technical barriers encompassed fundamental physics and engineering limitations. Electromagnetic navigation faced interference from metallic implants and field distortion requiring exclusion criteria [15]. Near-infrared fluorescence depth penetration limitations restricted application to superficial lesions, with signal degradation from blood and cauterised tissue [2,23,26]. Inductive sensors encountered detection range constraints (<4 mm) and electromagnetic field limitations [9,10]. Image-guidance systems struggled with liver deformation, producing 5–7 mm registration errors and posterior segment challenges [24]. Ultrasound-based approaches required lesion visibility and optical line-of-sight maintenance [18]. Mobile organ displacement compromised navigation accuracy [17].

*Transferable/Adjacent Studies*

The most informative transferable contribution to adoption potential assessment concerns regulatory readiness and physics-based technical barriers. RFID localisation systems have achieved clinical regulatory clearance through breast and lung applications [7,12,37], establishing a regulatory precedent that substantially reduces barriers for colorectal adaptation compared to technologies still at investigational stage; the magnetically stabilised injectable hydrogel [13] represents the opposite end of this spectrum, requiring new regulatory approval pathways for its novel biomaterial composition. Regarding scalability, multiple investigator groups in adjacent specialties explicitly identified colorectal surgery as a target domain for their localisation technologies [7,32,37,40,41], reflecting a recognised translational trajectory rather than speculative extrapolation. However, physics-based technical barriers documented in transferable applications may prove more restrictive in colorectal contexts than in their original settings: the RFID detection range of 30 mm confirmed adequate for breast tissue [37] may be insufficient for larger abdominal cavities or deeply seated colorectal lesions; the magnetic seed depth limitation of 3.5 cm [11] may disproportionately affect obese patients undergoing colorectal surgery; and RFID-related MRI signal artifacts [12] carry particular relevance in rectal cancer management where neoadjuvant chemoradiotherapy and treatment response monitoring by MRI are standard practice. Compatibility with robotic surgical platforms was confirmed for both RFID [37] and near-infrared fluorescent clip systems [40] in clinical series, directly relevant to the expanding use of robotic approaches in colorectal surgery.

#### 3.3.8. Evidence Quality and Risk of Bias

Study design hierarchy among the 18 directly applicable investigations demonstrated substantial heterogeneity. Only one randomised controlled trial achieved Level I evidence, examining electromagnetic navigation for retroperitoneal lymph node dissection [15]. Prospective clinical trials without randomisation included image-guidance systems [18,24] and optical stereotactic navigation [17]. Retrospective cohort studies examined thermal ablation modalities [22], surgical timing strategies [25], and near-infrared fluorescence imaging [2], with several employing propensity score matching [1,25]. Lower evidence levels included preclinical laboratory and animal studies [9,10], ex vivo experimental designs [10], and case series [6]. The predominance of non-randomised designs (94.4%, 17/18 studies) substantially constrained evidence strength.

Sample sizes varied considerably. The sole RCT enrolled 69 patients [15]. Prospective trials ranged from 25 to 50 patients [18,24]. Retrospective series spanned 86–336 patients [2,25]. Preclinical investigations employed limited human specimen validation alongside animal models [9]. Sample size calculations were reported infrequently.

Comparative study designs against current standards were uncommon. The electromagnetic navigation RCT compared against conventional surgery [15]. Propensity-matched retrospective analyses contrasted surgical timing strategies [25] and localisation approaches [1]. Near-infrared fluorescence imaging compared detection rates between NIRF-guided and standard surgery [2]. Nanoparticle studies compared targeted versus non-targeted formulations [27]. Most early-phase technology development studies employed single-arm designs without contemporaneous controls [6,9,17]. Standardised metrics for outcome assessment varied across investigations, limiting cross-study comparisons.

Risk of bias for all randomised controlled trials identified across the 89 included studies was assessed using the Cochrane RoB 2 tool; results are presented in Table 3. Ten studies met the pre-specified ROBINS-I selection criteria: five directly applicable colorectal clinical studies [1,2,18,24,25], three key transferable cohort studies included for their contribution to technology group evidence synthesis [7,11,12], and two additional studies [33,42] that were initially classified as RCTs in preliminary screening but reclassified as non-randomised following full-text assessment. Results are presented in Table 4. Among the ten non-randomised studies assessed with ROBINS-I, four were judged to have serious risk of bias predominantly in Domain 1 (confounding): [1,7,25,42]. In the first three, treatment or technique allocation was determined by clinician or institutional discretion rather than randomisation, with residual baseline imbalances persisting after propensity-score matching in [1,25]. In [42], comparison with a historical fluoroscopy cohort introduced both selection bias and temporal confounding. Findings from these four studies should be interpreted as hypothesis-generating rather than as evidence of superiority of one approach over another.

Certainty of evidence was assessed using the GRADE approach for the four technology groups meeting the pre-specified synthesis threshold—ICG/NIR fluorescence, electromagnetic navigation, RFID localisation, and magnetic seed localisation; results are presented in Table 5.

## 4. Discussion

### 4.1. Principal Findings and Clinical Significance

This scoping review identified 89 studies examining radiofrequency, electromagnetic, and alternative identification methods for enhanced laparoscopic detection of small colorectal tumours, with 18 studies directly applicable to colorectal surgery and 71 demonstrating transferable technologies from related anatomical domains. Principal findings revealed substantial heterogeneity in technological approaches, detection performance, and evidence quality. Detection performance varied substantially across modalities and outcome definitions—ranging from 16% for sonographically occult disappearing liver metastases using image guidance [18] to 85–97% for complete lymph node removal with electromagnetic navigation [15] and 85–90% for clip detection in controlled ex vivo settings [9]—with these figures reflecting heterogeneous endpoints in distinct clinical contexts rather than equivalent comparative metrics. Localisation precision varied from millimeter-scale accuracy for contact-based sensors with millimeter-scale discrimination capability [9,10] to depth-measurement capabilities for radioguided approaches [19]. Near-infrared fluorescence imaging demonstrated significantly higher tumour-to-background contrast versus normal tissue [27] and enabled detection of significantly smaller lesions (3.2 ± 1.8 mm) compared to standard techniques [2], while thermal ablation achieved successful treatment in 92–93% of lesions [22].

Clinical significance extends beyond detection metrics to surgical outcome improvements. Electromagnetic navigation achieved 85% complete removal of targeted retroperitoneal lymph nodes compared to 50% with conventional techniques [15], suggesting potential for margin adequacy enhancement in colorectal resections. Image-guidance systems enabled identification of previously undetectable disappearing metastases in 16% of cases [18], potentially reducing futile laparotomies and enabling more complete oncologic resections. However, a substantial gap persists between current standard practice—predominantly colonoscopic tattooing with India ink [1]—and emerging technological solutions. Tattooing faces challenges including unpredictable dye migration, clip dislodgement risk, and difficulty localising small lesions without visible serosal changes [1], while offering no real-time intraoperative guidance. The reviewed technologies address these limitations through various mechanisms, yet none has achieved widespread clinical adoption, reflecting persistent barriers in cost, workflow integration, and evidence maturity.

### 4.2. Technology-Specific Insights and Comparative Analysis

The following comparative analysis draws on the technology-level synthesis presented in Supplementary Table S2 (Figure 2).

RFID technologies from breast applications demonstrated high detection rates with favourable safety profiles, showing post-operative complications in 1.7% of cases primarily comprising minor seromas and wound infections [12]. Clinical readiness appeared advanced with FDA-approved systems and established workflows. However, RFID tag localisation was significantly more likely to fail than wire-guided localisation in a retrospective cohort of 680 breast cancer patients, with failed tags requiring subsequent wire placement [7]. This observed failure rate—attributed to tags relying on friction without anchoring—raises additional concerns for colorectal adaptation, where mobile bowel subjected to peristalsis and laparoscopic manipulation would further increase migration and dislodgement risk. Colorectal-specific validation of tag stability is therefore essential before clinical adoption.

Magnetic seed systems demonstrated 100% localisation success without migration [11], though detection was limited to 3.5 cm depth. RFID systems offered pragmatic near-term implementation potential given technological maturity and workflow compatibility with existing CT-guided marking procedures [6].

Electromagnetic and magnetic tracking systems exhibited divergent characteristics. Electromagnetic navigation platforms [15,17] provided sub-millimeter spatial resolution (0.7 mm median registration error) [17] and real-time 3D visualisation, facilitating complex anatomical dissections. However, substantial capital costs (USD 200,000–400,000 for navigation platforms, and USD 300,000–500,000 for 3D C-arm imaging) [17], extensive training requirements, and susceptibility to metallic instrument interference limited accessibility [15]. Magnetic seed technologies [11] offered simpler workflows with 100% localisation success and no migration, though 3.5 cm detection depth constrained applications to superficial lesions. Inductive proximity sensors [9,10] represented innovative electromagnetic detection principles with millimeter-scale precision (5.07 mm discrimination) [10], but remained in prototype stages requiring clip modifications and tissue penetration optimisation. Magnetic approaches demonstrated favourable ergonomics and minimal interference, while electromagnetic navigation demanded workflow restructuring and dedicated technical support [15,17].

Optical fluorescence methods divided into non-targeted and targeted approaches. Non-targeted ICG administration [2] provided minimal per-patient cost, seamless integration with existing laparoscopic fluorescence cameras, and minimal training requirements (1–2 cases). However, non-specific biodistribution and limited 8 mm tissue penetration constrained detection to exposed or superficial lesions [2]. Targeted fluorescent probes, including receptor-specific agents (Cytalux for folate receptor-alpha) [29] and peptide-functionalised nanoparticles demonstrating 4.0-fold higher tumour fluorescence versus normal tissue [27], enhanced tumour specificity, but faced substantial costs (Cytalux ~USD 5000, compared to ICG USD 50–200) [29] and complex pharmacokinetics requiring optimised administration timing. FDA-approved fluorophores (ICG, Cytalux, Gleolan) [29] demonstrated clinical readiness advantages over investigational nanoparticle systems [27]. Optical methods excelled in real-time visual feedback and ergonomic integration but suffered from 8–10 mm depth penetration limitations precluding detection of deeply positioned or buried lesions [2,29].

Hybrid and emerging approaches combined complementary modality strengths. Radioactive-fluorescent dual tracers [19] integrated gamma probe sensitivity for deep tissue detection with fluorescence precision for surface guidance, though radioactive handling requirements and regulatory complexity impeded adoption. Image-guidance systems combining preoperative CT/MRI with intraoperative ultrasound [18,24,30] addressed tissue deformation through real-time registration, but demanded substantial capital investment [24] and computational resources. Deep learning-assisted segmentation [17] streamlined workflow compared to manual approaches, representing promising integration of artificial intelligence. Augmented reality overlays [17,26] offered intuitive visualisation but required validation in dynamic surgical fields with tissue deformation.

Comparing technologies across evaluation domains revealed divergent performance profiles with no universal solution. Electromagnetic navigation excelled in precision and 3D guidance but imposed high costs and complexity. RFID and magnetic systems balanced performance with pragmatic implementation but faced depth limitations (3.5 cm for magnetic seeds). Optical fluorescence provided elegant integration and real-time feedback but suffered tissue penetration constraints (8 mm depth). Hybrid approaches combined strengths but amplified complexity and cost. Technology selection likely depends on clinical context. Electromagnetic navigation’s precision suits complex dissections but requires substantial infrastructure. RFID/magnetic systems offer implementation advantages for routine applications. Optical fluorescence provides accessible real-time guidance for surface assessment. However, head-to-head comparative trials are needed to validate context-specific selection strategies.

### 4.3. Practical Implementation Challenges

Technical barriers encompassed fundamental physics limitations and engineering constraints. Tissue penetration differences between modalities remained a fundamental constraint, as detailed in Section 3.3.2. RFID detection range was not systematically quantified in breast applications [7,12]; the 30 mm maximum range confirmed in thoracic applications [37] may prove inadequate for larger abdominal cavities and deeper colorectal lesions, representing a more restrictive constraint than encountered in breast or pulmonary contexts. Electromagnetic tracking accuracy degraded with distance from field generators and in the presence of ferromagnetic instruments, requiring patient exclusion criteria for metallic implants [15], while inductive sensors required optimisation of resonance frequencies to minimise environmental interference [9,10]. Sterilisation compatibility posed challenges for reusable detection probes, with some RFID and magnetic systems requiring single-use components increasing per-case costs [7,11,12]. Respiratory motion and tissue deformation during insufflation reduced navigation accuracy, necessitating continuous registration updates [15,17] or deformable image registration algorithms with computational processing demands [35].

Workflow integration challenges extended beyond technical functionality to practical surgical coordination. Electromagnetic navigation required CBCT scanning and calibration setup, though total operative time was not significantly prolonged [15]. Preoperative marker placement via colonoscopy [1] or CT guidance [6] required multidisciplinary coordination and added patient visits, increasing logistical complexity. Intraoperative learning curves ranged from minimal training (1–2 cases) for fluorescence imaging [2] to more extensive surgeon training for complex navigation systems [15], demanding institutional commitment to competency development. Operating room footprint expanded with additional equipment (navigation workstations, optical tracking cameras, specialised probes), challenging space-constrained environments. Integration with robotic surgical platforms showed promise for fluorescence imaging with da Vinci systems [2,29] but remained underdeveloped for electromagnetic and RFID modalities.

Economic considerations extended beyond initial purchase costs to include total cost of ownership. High-end navigation systems required significant infrastructure investment and ongoing costs for software licensing, technical support, and equipment servicing [15], achievable primarily in high-volume tertiary centres. Per-case disposable costs ranged from minimal (tattooing dye ~USD 20–50, clips ~USD 100–200 [1]) to considerable per-patient costs for electromagnetic tracking [15]. However, comprehensive cost-effectiveness analyses remained sparse, with most studies reporting acquisition costs without accounting for training expenses, maintenance, or potential savings from reduced operative time, decreased complications, or avoided reoperations; formal cost-effectiveness modelling specific to colorectal tumour localisation incorporating quality-adjusted outcomes and full pathway costs is entirely absent from the current literature, in contrast to adjacent specialties where cost-offset models for wireless localisation technologies have been developed [7,12,32]. Transferable technology economic data from breast cancer applications demonstrated potential cost benefits for RFID and radar-based systems through reduced reoperation rates, with electromagnetic chip systems avoiding approximately 21 re-excisions per 100 annual cases [32], suggesting similar analysis is warranted for colorectal applications.

From a healthcare system perspective, the cost data summarised in Table 2 suggest a stratification of technology adoption by institutional resource tier. Near-infrared fluorescence systems, with capital costs of USD 30,000–300,000 and per-patient costs of USD 100–300 [2,29], fall within an order of magnitude consistent with adoption across secondary as well as tertiary centres already equipped with laparoscopic or robotic platforms supporting fluorescence imaging. By contrast, electromagnetic and optical stereotactic navigation systems, with combined navigation and 3D C-arm infrastructure costs reported up to USD 400,000–900,000 [17], alongside ongoing software licensing and technical support requirements [15], are realistically confined to high-volume academic centres able to amortise these costs across multiple surgical indications beyond colorectal localisation. This stratification implies that no single technology is likely to achieve uniform adoption across the diverse range of institutions performing colorectal cancer surgery, and that future technology selection guidance should explicitly account for institutional resource tier alongside clinical performance.

Regulatory and reimbursement pathways created substantial adoption barriers. Novel targeting agents including peptide-functionalised nanoparticles [27] required regulatory approval through investigational new drug applications and clinical trials, while receptor-specific probes faced lengthy regulatory pathways [29]. Device regulatory approval for RFID and magnetic systems required safety and efficacy documentation [11]. Reimbursement uncertainties deterred institutional investment, as many advanced localisation technologies faced evolving reimbursement structures and unclear pathways for novel agents [27,29]. This may create a barrier to adoption: cost-effectiveness evidence requires widespread use, yet payer coverage often depends on such evidence. Where wireless localisation technologies have achieved sufficient adoption in non-colorectal surgical practice to establish billing and reimbursement precedents, this experience could provide a template for expediting health system adoption pathways in colorectal applications. Conversely, technologies without such precedent—including investigational targeted fluorescent probes and inductive proximity sensor platforms—face a compounded barrier requiring simultaneous regulatory approval and novel reimbursement code development. Mapping reimbursement pathway status was not a formal extraction domain in this review but represents an important component of future health technology assessments in this field.

### 4.4. Evidence Quality and Research Gaps

Certainty of evidence assessed using the GRADE approach was Low for electromagnetic navigation—the sole technology supported by a phase-3 RCT—and Very Low for ICG fluorescence, RFID localisation, and magnetic seed localisation, primarily reflecting the absence of colorectal-specific clinical validation, heterogeneous outcome definitions, and predominantly observational study designs. GRADE assessment was not extended to the remaining five technology groups, as the available evidence comprised predominantly preclinical studies, phantom validations, or heterogeneous case series without extractable common outcomes, rendering formal certainty grading uninformative beyond the narrative appraisal already provided. These findings formally confirm that the evidence base for all currently available tumour localisation technologies in colorectal surgery remains insufficient to support definitive clinical recommendations, and underscore the urgent need for prospective colorectal-specific trials.

Publication bias represents a particular concern for the preclinical and early-phase feasibility literature comprising the majority of this review’s corpus. The GRADE-based evidence maturity characterisation flagged publication bias as suspected for three of the four technology groups formally evaluated (Table 5), reflecting a structural tendency for positive feasibility results to reach publication while unsuccessful prototype iterations, failed detection attempts, or negative comparative findings remain unpublished. This concern is compounded by the predominance of single-arm, inventor-led studies, in which the research group developing a technology also conducts and reports its initial evaluation, potentially introducing optimism bias in study design, outcome selection, and result interpretation. This consideration applies directly to two studies included in this review for which co-authors of the present manuscript are also co-authors of the primary research [9,10], as declared in the Conflicts of Interest statement; the structured seven-domain assessment and ROBINS-I appraisal applied uniformly across all included studies were intended to mitigate, though not eliminate, this risk.

Preclinical and ex vivo studies carry additional translational limitations beyond publication bias. Benchtop and ex vivo specimen testing—employed for inductive proximity sensors [9,10], targeted nanoparticle probes [27], and several optical imaging studies [23,43,44]—cannot replicate key features of the in vivo surgical environment, including pneumoperitoneum-induced tissue deformation, bowel peristalsis, perfusion-dependent tissue optical properties, and the time pressure and ergonomic constraints of live surgery. Detection rates and precision measurements obtained under controlled laboratory conditions, frequently performed by the device’s own developers, may not generalise to multi-operator clinical use across diverse patient anatomies. Animal models, such as the rabbit pulmonary fluorescence localisation study included in this review [45], partially bridge this gap but introduce species-specific anatomical differences that limit direct extrapolation to human colorectal anatomy. These factors represent an important caveat when considering the performance ranges summarised in Section 3.3.2, and highlight the absence of multi-centre, multi-operator validation studies as a significant gap in the current evidence base.

Evidence quality assessment revealed predominance of early-phase investigational studies with limited high-level evidence. Among 18 primary studies directly examining colorectal applications, only one RCT [15] provided Level I evidence, with the remaining 17 employing non-randomised designs. Four additional systematic reviews and narrative reviews synthesised existing evidence [23,26,28,29]. Preclinical and ex vivo investigations [9,10,27] provided proof-of-concept but lacked clinical validation. Small sample sizes in pilot studies [6,17,30] precluded adequate statistical power for detecting clinically meaningful differences. Most studies represented single-institution experiences, limiting generalisability across diverse patient populations, surgical techniques, and institutional resources. Retrospective designs introduced selection and detection biases despite propensity matching attempts [1,2,25].

Lack of standardised outcome metrics limited the potential for robust cross-study comparisons and meta-analytic synthesis. Detection rate definitions varied from percentage of successfully identified markers [9,10] to complete resection achievement [15] to lesion localisation success [18], precluding quantitative pooling. Precision metrics employed heterogeneous measurement approaches including mean error distances [9,10], probability calculations [15], and subjective assessments [17]. Safety reporting ranged from structured Clavien-Dindo classification [22] to narrative descriptions [2,6], limiting complication rate comparisons. Economic evaluations predominantly reported acquisition costs without standardised cost-effectiveness or cost-utility frameworks. User experience assessments employed non-validated questionnaires rather than established instruments [10,15]. Development and validation of standardised outcome sets specific to colorectal tumour localisation technologies represents a critical research priority.

Standardisation of these heterogeneous definitions—including the distinction between localisation accuracy (closeness to the true target position) and localisation precision (reproducibility of the measurement), which are frequently conflated in the literature—represents the necessary first step toward a usable core outcome set. Building on this gap, a minimum core outcome set for future colorectal tumour localisation studies could be structured around three tiers. Primary outcomes should include: detection rate, defined explicitly as the proportion of placed markers or targeted lesions successfully identified intraoperatively prior to resection, with the denominator (per-patient versus per-lesion) and detection method stated; localisation precision, reported as the linear distance in millimetres between the marker or detection signal and the tumour centre or resection margin; and technical placement success, reported separately from intraoperative detection to distinguish marker deployment failures from detection failures. Secondary outcomes should include marker migration rate over a defined follow-up period, marking-to-surgery interval and its tolerance window, adverse events classified using a structured instrument such as the Clavien-Dindo classification, R0 resection rate, and conversion to open surgery rate. Exploratory outcomes relevant to health system adoption should include total operative time attributable to the localisation procedure, reoperation rate within a defined interval, and cost per successful intraoperative detection. Formal development and validation of this proposed framework through established core outcome set methodology—involving a systematic review of existing outcome measures followed by a Delphi consensus process with colorectal surgeons, interventional radiologists, endoscopists, medical physicists, and patient representatives, consistent with COMET Initiative guidance—would substantially improve the comparability of future evidence and represents a priority companion project to this scoping review.

Limited head-to-head comparative trials represented an important evidence gap. While some studies compared technologies against standard care [2] or between formulation variants [27], most technology development studies employed non-randomised designs without contemporaneous controls [6,9,17]. The sole RCT contrasted electromagnetic navigation against conventional surgery [15] but did not evaluate alternative advanced localisation methods. Direct head-to-head comparisons between RFID, magnetic, optical, and electromagnetic approaches would elucidate relative advantages, cost-effectiveness, and optimal clinical contexts for each technology. Multi-arm trials incorporating patient-reported outcomes, quality of life metrics, and long-term oncologic endpoints alongside traditional detection performance measures would provide comprehensive evidence for clinical decision-making and guideline development.

Long-term safety data gaps included marker retention, migration, and chronic tissue reactions beyond typical 30–90 day follow-up periods. Metallic clips remain permanently in tissue with theoretical migration risk requiring systematic long-term imaging surveillance [6]. Nanoparticle clearance through metabolism by liver Kupffer cells and spleen [27] required extended monitoring for potential accumulation effects. Radioactive seed retention, when employed for localization, necessitated long-term radiation exposure assessment given minimal but persistent tissue exposure [19]. Tattoo permanence [1] lacked systematic evaluation of chronic inflammatory responses or oncologic implications from dye deposition in lymphatic drainage pathways, as long-term follow-up focused on oncologic rather than marker-related outcomes.

### 4.5. Transferability to Colorectal Applications

Anatomical and mechanical compatibility of transferable technologies with colorectal applications varied substantially across modalities. RFID tag systems developed for pulmonary nodule and breast lesion localisation demonstrated adequate detection through breast tissue depths [7,12], though specific detection ranges were not quantified. However, bowel wall thickness (2–4 mm) and peritoneal fat distribution differed from breast and lung parenchyma, potentially affecting signal propagation. Magnetic seed technologies functioned effectively at depths up to 3.5 cm in breast tissue [11] without observed migration, suggesting adequate range for subserosal colorectal lesions, though colonic peristalsis and laparoscopic manipulation introduce mechanical forces absent in breast applications. Wire-based localisation techniques exhibited potential for dislodgement and migration in pulmonary procedures [32,34], raising questions about stability in mobile bowel segments subjected to insufflation and manipulation during laparoscopy.

Electromagnetic tracking systems validated in pulmonary navigation [31] employed similar electromagnetic detection principles as colorectal inductive sensor platforms [9,10], suggesting technological transferability. However, thoracic applications faced significant tissue deformation between imaging and operative states [31], while abdominal organs exhibited both respiratory and peristaltic motion requiring continuous registration updates. Radar-based wireless localisation demonstrated effectiveness for breast tissue localisation [32], potentially offering marker stability advantages for colorectal applications, though specific detection ranges were not quantified and utility for deeply positioned or thick-walled lesions requires validation.

Workflow integration feasibility demonstrated variable compatibility with existing colorectal surgical paradigms. Injectable fluorescent tracers employed in navigational bronchoscopy [33] aligned closely with established colonoscopic tattooing workflows [1], requiring minimal protocol modifications beyond tracer selection and timing optimisation. Preoperative RFID tag placement under ultrasound or CT guidance [7,12] paralleled existing practices for CT-guided clip placement [6], though radiofrequency detection probes necessitated integration into laparoscopic instrument sets. Magnetic seed localisation workflows [11] required coordination between interventional radiologists for placement and surgeons for detection, similar to multidisciplinary coordination already established for neoadjuvant therapy monitoring [25,28].

Deformable image registration software validated in hepatic thermal ablation [35] demanded substantial computational infrastructure and preoperative imaging segmentation, potentially challenging workflow integration in time-sensitive emergency colorectal procedures. However, elective oncologic resections with planned neoadjuvant therapy allowed adequate preparation time. Electromagnetic navigation systems from thoracic applications [31] required operating room setup and registration procedures comparable to systems already evaluated in colorectal contexts [15,17], suggesting feasible workflow incorporation with appropriate training investments.

Required adaptations and transfer potential rankings identified several promising candidates. High transfer potential technologies included: (1) RFID tag systems, requiring minimal adaptation beyond tag placement technique optimisation and probe integration into laparoscopic ports [7,12]; (2) Injectable fluorescent tracers (ICG:albumin formulations), necessitating only endoscopic injection protocol development given existing fluorescence imaging infrastructure [33]; (3) Magnetic seed localisation, requiring validation of marker stability in mobile bowel and detection probe ergonomics during laparoscopy [11]. Moderate transfer potential technologies encompassed: (1) Radar-based wireless localisation, requiring validation of detection range adequacy through bowel wall and mesenteric fat [32]; (2) Electromagnetic tracking systems with embedded position sensors, necessitating marker miniaturisation and validation of motion compensation algorithms for peristaltic activity [31]; (3) Deformable image registration software, requiring optimisation for abdominal organ deformation patterns and real-time computational performance [35].

Lower transfer potential technologies included wire-based localisation techniques due to migration and dislodgement risks in mobile anatomy [5,32,34], and complex multimodal navigation platforms requiring extensive capital investment (USD 500,000–2,000,000 for hybrid operating rooms) and workflow restructuring [34]. Hybrid approaches combining high-transfer-potential modalities warrant investigation. Examples could include RFID tags with fluorescent coatings or magnetic seeds with radiopaque markers to achieve synergistic detection capabilities. Transferable technology assessment indicated that adaptation complexity ranged from minimal protocol modifications (injectable tracers) to substantial engineering development (miniaturised electromagnetic sensors), with intermediate options (RFID, magnetic seeds) representing pragmatic near-term implementation candidates.

### 4.6. Future Directions and Clinical Translation

Integration with artificial intelligence, robotics, and augmented reality represents transformative future directions. AI-assisted automated tumour segmentation from preoperative imaging [17] could streamline navigation workflow preparation, with deep learning already demonstrating a reduction in manual segmentation time from 12 h to approximately 4 h and potential for further optimisation. Machine learning algorithms for real-time fluorescence signal quantification and background correction [27] may enhance tumour margin discrimination beyond subjective visual assessment. Predictive modelling for disappearing metastasis risk stratification [28] could guide selective marker placement in high-risk lesions, optimising resource utilisation. Robotic surgical platforms with native fluorescence imaging integration [2,29] facilitate optical method adoption, while future electromagnetic and RFID compatibility development would expand technological accessibility. Near-infrared fluorescent marking clips specifically engineered for robotic gastrointestinal surgery with da Vinci Firefly compatibility have recently demonstrated clinical feasibility in a gastric cancer cohort [40], representing a directly relevant translational step for robotic colorectal platforms. Augmented reality head-mounted displays projecting tumour locations onto surgeon visual fields [17,26] could revolutionise intraoperative guidance, though validation in dynamic surgical environments with tissue deformation remains essential.

Standardisation efforts and guideline development would benefit from coordinated multidisciplinary initiatives. Consensus conferences convening colorectal surgeons, interventional radiologists, medical physicists, and industry representatives could establish standardised outcome definitions, safety reporting frameworks, and comparative effectiveness trial designs. Professional societies (ASCRS, ESCP, SAGES) could develop evidence-based guidelines for appropriate technology selection based on lesion characteristics, institutional resources, and clinical contexts. Efforts toward regulatory harmonisation across jurisdictions may accelerate device approval and market access, while collaborative reimbursement frameworks linking payment to demonstrated outcome improvements could incentivise adoption of validated technologies.

Promising technologies closest to clinical adoption include RFID tag systems given established regulatory pathways, successful breast/pulmonary implementation, and defined workflows [7,12]; ICG-based near-infrared fluorescence given minimal cost (USD 100–300 per patient), widespread camera availability, and favourable safety profiles with extremely rare adverse reactions [2,29]; and magnetic seed localisation given established workflows and elimination of nuclear medicine requirements [11]. These technologies warrant priority investigation through multicenter comparative effectiveness trials in colorectal applications. Research priorities for the near future encompass: (1) head-to-head RCTs comparing RFID, magnetic, and optical approaches; (2) long-term safety and oncologic outcome registries; (3) cost-effectiveness analyses incorporating quality-adjusted life years; (4) workflow optimisation studies identifying implementation barriers; (5) validation of transferable technologies (RFID, magnetic seeds, injectable fluorescent tracers) specifically in colorectal anatomy and surgical contexts. Addressing these priorities will accelerate evidence-based integration of advanced localisation technologies into routine colorectal surgical practice.

## 5. Conclusions

### 5.1. Principal Findings

This scoping review of 89 studies examining radiofrequency, electromagnetic, and alternative identification methods for laparoscopic colorectal tumour detection revealed substantial technological diversity with variable evidence maturity. Among 18 primary studies directly applicable to colorectal applications, detection performance varied substantially by modality and outcome definition, ranging from 16% for sonographically occult disappearing metastases to 85–97% for complete target removal with electromagnetic navigation. Electromagnetic navigation and inductive proximity sensors demonstrated highest precision (millimeter-scale accuracy) but required significant infrastructure investments. Near-infrared fluorescence imaging offered pragmatic integration with existing laparoscopic systems at minimal incremental cost, though tissue penetration limitations (8–10 mm) constrained application to superficial lesions. RFID and magnetic seed technologies from transferable pulmonary and breast applications demonstrated high detection rates (91.9–100%) in several studies [7,11,12] and favourable safety profiles, representing promising adaptation candidates for colorectal contexts pending validation of marker stability in mobile bowel anatomy.

### 5.2. Clinical Implications

No single technology currently addresses all clinical requirements for enhanced colorectal tumour localisation. Electromagnetic navigation systems suit complex anatomical dissections in well-resourced tertiary centres, while RFID and magnetic approaches offer balanced performance for routine lesion localisation in community settings. Near-infrared fluorescence provides universal applicability for surface tumour margin assessment across all practice environments. The substantial gap between current standard practice (colonoscopic tattooing) and emerging technologies reflects persistent barriers in cost, workflow integration, and evidence quality rather than technological inadequacy. Technology selection is likely to depend on institutional capabilities, case complexity, and lesion characteristics, with hybrid approaches combining complementary modalities representing a potential avenue to optimise detection performance.

### 5.3. Research Priorities

Critical research needs include head-to-head randomised controlled trials comparing RFID, magnetic, optical, and electromagnetic approaches; development and validation of standardised outcome metrics enabling cross-study synthesis; long-term safety registries documenting marker retention, migration, and chronic tissue reactions; comprehensive cost-effectiveness analyses incorporating quality-adjusted outcomes; and focused validation studies adapting high-potential transferable technologies (RFID tags, magnetic seeds, injectable fluorescent tracers) to colorectal surgical contexts.

### 5.4. Limitations

This review’s limitations include predominance of early-phase non-randomised studies (94.4% of primary studies), limiting evidence strength; heterogeneous outcome definitions limiting cross-study comparability; potential publication bias favouring positive feasibility results (Section 4.4); incomplete economic data across most technologies; and reliance on transferable technology extrapolation without direct colorectal validation.

### 5.5. Final Statement

While multiple promising technologies demonstrate potential to enhance laparoscopic colorectal tumour detection beyond conventional tattooing, rigorous comparative effectiveness research and standardisation efforts are essential to guide evidence-based clinical adoption and optimise patient outcomes.

## 6. Studies Included in This Review

Characteristics of 89 studies included in the review. RCT = randomised controlled trial; EM = electromagnetic; RFID = radiofrequency identification; NIR = near-infrared; ICG = indocyanine green; US = ultrasound; NR = not reported.

**Table 1:** Complete list of studies included in this review.

Application	Technology/Modality	Study Design/n	Study
Colorectal	Electromagnetic navigation with tracking	RCT, n = 69	[15]
Colorectal	Image guidance system (Explorer) using tracked ultrasound with optical navigation to locate radiographically occult colorectal liver metastases that disappeared during chemotherapy treatment	Prospective, n = 25	[18]
Colorectal	Inductive proximity sensor	Preclinical, n = 25	[9]
Colorectal	Inductive proximity sensors detecting modified endoscopic hemostatic clips placed at tumour periphery, using electromagnetic field detection through laparoscopic instruments	Preclinical, n = NR	[10]
Colorectal	Ultrasound-guided thermal ablation using RFA (Cool-tip) and MWA (Emprint) electrodes for treating colorectal liver metastases during open surgery	Retrospective, n = 120	[22]
Colorectal	Near-infrared optical imaging with smart fluorescent probes, iron oxide nanoparticle-enhanced MRI, and cathepsin-activated fluorochromes for colorectal cancer detection and staging	Preclinical, n = NR	[23]
Colorectal	Ultrasound-guided localisation	Prospective, n = 14	[24]
Colorectal	Preoperative CT-guided placement of manually straightened McKenzie-Diener silver clips into small liver metastases before chemotherapy initiation, identified later during surgery with intraoperative ultrasonography	Case series, n = 3	[6]
Colorectal	Fiducial markers mentioned for liver metastasis marking before neoadjuvant therapy to mark lesions that may disappear radiologically but remain present; technique not primary focus of study	Retrospective, n = 336	[25]
Colorectal	Near-infrared fluorescence imaging	Review	[26]
Colorectal	Preoperative colonoscopic localisation via endoscopic tattooing (circumferential dye injection distal to tumour) or metallic clip placement (endoscopic hemoclip applied to mucosa adjacent to lesion)	Retrospective, n = 296	[1]
Colorectal	Optical stereotactic navigation using preoperative CT/MRI fusion with deep learning-assisted 3D segmentation of tumour and anatomical structures, tracked intraoperatively with calibrated pointer or laparoscopic instrument via infrared camera system	Prospective cohort, n = 10	[17]
Colorectal	Near-infrared fluorescence imaging	Preclinical, n = 3	[27]
Colorectal	Near-infrared fluorescence imaging using indocyanine green (ICG) administered 1–2 days pre-surgery to identify subcapsular colorectal liver metastases during open/laparoscopic resection	Retrospective, n = 173	[2]
Colorectal	Fiducial marker placement for disappearing colorectal liver metastases (DLMs); discusses imaging modalities (Gd-EOB-DTPA MRI, CE-IOUS) and augmented reality navigation for intraoperative lesion localisation when tumours vanish after chemotherapy	Review	[28]
Colorectal	Near-infrared fluorescence imaging	Case series, n = 23	[29]
Colorectal	Radioguided surgery	Review	[19]
Colorectal	US/MRI fusion guidance using pre-chemotherapy MRI as reference; employs anatomical landmarks (aorta, hepatic veins, portal branches) for volume matching without physical markers	Case series, n = 1	[30]
Breast	Wire localisation	Retrospective, n = 177	[46]
Lung	Intraoperative lesion marking performed indirectly through robotic laser guidance, calibrated to preoperative CT data	Feasibility study, n = 4	[47]
Liver	CT-guided percutaneous thermal ablation using electromagnetic navigation system (EMNS)—Imactis^®^ (BVM Medical, Grenoble, France) with real-time needle trajectory display in two perpendicular planes	Retrospective, n = 93	[16]
Lung	Electromagnetic navigational bronchoscopy (ENBL) with CT-guided virtual bronchoscopy mapping followed by bronchoscopic dye injection into/adjacent to nodule	Retrospective, n = 51	[48]
Other	Nanocarbon suspension injection combined with metal coils for precise localisation of metastatic lymph nodes during laparoscopic surgery, guided by preoperative 68Ga-DOTA-NOC PET-CT imaging	Case series, n = 1	[49]
Lung	Intraoperative molecular imaging using pafolacianine, a folate receptor-targeted fluorescent agent visualised with near-infrared imaging system	Retrospective, n = 39	[50]
Liver	Optical tracking system with navigated ultrasound combining preoperative CT/MRI with real-time instrument tracking and 3D visualisation models	Prospective, n = 50	[51]
Other	Augmented reality platform with head-mounted display using electromagnetic tracking and 3D holographic projections overlaid onto patient anatomy	Observational, n = 12	[52]
Other	Near-infrared fluorescent probes for tumour and lymph node visualisation, divided into passive targeting (tumour-directed) and active targeting (lymph node-specific) approaches	Preclinical, n = NR	[43]
Lung	Multiple techniques including fiducials (hookwires, microcoils), injectable dyes (ICG, methylene blue, Tc99m), robotic bronchoscopy, electromagnetic navigation, and 3D planning systems	Review	[36]
Liver	Manual and automatic registered volume navigation (mVNav/aVNav) coupling real-time US with pre-interventional CT/MR imaging for radiofrequency and microwave ablation of liver lesions	Retrospective, n = 25	[53]
Breast	Stereotactic radiofrequency ablation using optical navigation system with three-dimensional planning and real-time image fusion for precise probe placement	Retrospective, n = 26	[54]
Breast	Comparison of wire-guided localisation (WGL) versus four non-radioactive seed technologies (Magseed^®^, Pintuition^®^, SAVI SCOUT^®^, LOCalizer™) for breast lesion marking.	Observational, n = 66	[38]
Liver	Near-infrared fluorescence imaging	Case series, n = 2	[4]
Breast	Magnetic seed localisation	Retrospective, n = 114	[55]
Other	Wire localisation	Review	[21]
Liver	Ultrasound-guided localisation	Case series, n = 1	[56]
Other	Radioguided surgery	Prospective, n = 12	[57]
Liver	Thermal ablation	RCT, n = 100	[35]
Other	Near-infrared fluorescence imaging	Case series, n = 1	[58]
Other	Augmented reality (AR) superimposes 3D reconstructed virtual images from CT/MRI onto surgical field using video-based, projection-based, or see-through displays	Review	[59]
Other	Endoscopic submucosal injection of a magnetically stabilised injectable hydrogel (MagLabel-IH) near lesion; intraop electromagnetic sensor array + handheld probe (SEML) dynamically register magnetic coordinates to anatomy for real-time localisation	Preclinical, n = NR	[13]
Lung	CT-guided percutaneous placement of fiber-coated platinum microcoils with pleural-tagging; coil tip exposed at pleura for direct thoracoscopic visualisation—allows VATS without intraop fluoroscopy.	Retrospective, n = 147	[60]
Lung	Intraoperative molecular imaging (IMI) using LS301 fluorescent agent administered 1+ days pre-surgery, visualised during minimally invasive/robotic surgery with near-infrared (NIR) imaging devices	Observational, n = 11	[61]
Other	Near-infrared fluorescence imaging	Prospective, n = 25	[3]
Lung	Near-infrared fluorescence imaging	Preclinical, n = 11	[45]
Lung	Near-infrared fluorescence imaging	Preclinical, n = 3	[62]
Liver	Electromagnetic tracking system (Aurora) with magnetic field generator pad under patient; sensors embedded in instrument tips track real-time 3D position of antenna and US probe	Prospective, n = 13	[63]
Breast	Intraoperative US-guided localisation using blue dye injection (n = 43) or guide-wire placement (n = 14); skin marking with pen to identify tumour location before surgical incision	Prospective, n = 57	[64]
Breast	RFID tag localisation	Retrospective, n = 680	[7]
Liver	Ultrasound-guided localisation	Prospective, n = 32	[65]
Other	RFID tag localisation	Retrospective, n = 10	[14]
Lung	Electromagnetic navigation with tracking	Retrospective, n = 10	[66]
Lung	Navigational bronchoscopy-guided transbronchial peritumoral ICG injection creating NIR “tattoo” for tumour localisation and sentinel lymph node mapping during VATS	Prospective pilot, n = 12	[33]
Liver	Augmented reality overlay of preoperative 3D CT model onto laparoscopic images using deformable registration; virtual tumour projection to liver surface via double projection system	Observational, n = 8	[67]
Lung	Robotic bronchoscopy-guided chemical localisation using ICG (indocyanine green) dye injection adjacent to lung nodules, visualised intraoperatively with near-infrared camera after robot docking	Prospective, n = 249	[41]
Other	Near-infrared fluorescence imaging	Review	[68]
Lung	Folate receptor-targeted near-infrared fluorescence imaging using pafolacianine combined with ultra-thin composite optical fiberscope for transbronchial tumour detection in real-time	Preclinical, n = 10	[69]
Other	Fluorescence-based optical imaging using Cy5.5-labelled albumin nanoparticles loaded with gold nanoclusters; photoacoustic imaging also used for gold detection in tumours after IV injection	Preclinical, n = 5	[44]
Other	Intraoperative fluorescence imaging using Fluobeam^®^700 portable device after IV injection of Angiostamp™700 (tumour-targeting αvβ3 integrin probe) 24 h pre-surgery for real-time nodule detection	Preclinical, n = 12	[70]
Other	Near-infrared fluorescence imaging	Preclinical, n = 3	[71]
Other	Near-infrared fluorescence imaging	Preclinical, n = 468	[72]
Other	Near-infrared autofluorescence microscopy (775 ± 50 nm excitation, 845 ± 55 nm emission) to identify autofluorescent substances in parathyroid/thyroid tissues without exogenous contrast agents	Observational, n = 7	[73]
Breast	Magnetic seed localisation	Prospective cohort, n = 32	[11]
Breast	SAVI SCOUT reflector system (radar-based) vs. standard wire localisation for non-palpable breast lesions during lumpectomy procedures	Retrospective, n = 84	[74]
Breast	Targeted axillary dissection using either ultrasound-guided gel-embedded clip wire localisation or magnetic surgical marker probe-guided localisation after neoadjuvant therapy	RCT, n = 42	[39]
Other	CT-guided preoperative placement of 3 mm biocompatible metal clip (fiducial) fixed on lamina or spinous process	Prospective cohort, n = 30	[42]
Breast	RFID tag localisation	Prospective, n = 299	[12]
Liver	Near-infrared fluorescence imaging	Review	[75]
Other	Wire localisation	Review	[76]
Lung	Near-infrared fluorescence imaging	Review	[5]
Other	Preoperative Tc-99m sulfur colloid SPECT/CT imaging with intraoperative handheld gamma probe detection, enhanced by augmented reality headset (HoloLens) for 3D visualisation	Preclinical, n = 8	[77]
Lung	Markerless approach using intraoperative CBCT imaging combined with preoperative CT registration and biomechanical modelling to compensate for lung deformation during pneumothorax	Retrospective, n = 5	[78]
Lung	Electromagnetic navigation with tracking	Prospective, n = 15	[79]
Breast	Preoperative bracketing using multiple localisers placed under imaging guidance (mammogram, ultrasound, MRI) for breast-conserving surgery of nonpalpable lesions requiring wide excision margins.	Retrospective, n = 118	[32]
Other	Thermal ablation	Retrospective, n = 34	[80]
Other	Near-infrared fluorescence imaging	Prospective, n = 13	[81]
Other	Near-infrared fluorescence imaging	Prospective, n = 9	[82]
Lung	Electromagnetic navigation with tracking	Review	[34]
Lung	Electromagnetic-tracked J-bar fiducial placed near nodule via needle insertion, with EM-sensorised surgical stapler providing real-time distance measurements	Prospective, n = 24	[31]
Lung	Extended reality hologram overlay of 3D-reconstructed CT anatomy onto thoracoscopic monitor	Prospective, n = 20	[83]
Lung	Electromagnetic navigation with tracking	Prospective, n = 75	[37]
Lung	Physics-based computational simulation predicting lung collapse deformation; virtual deflation algorithm with boundary conditions and contraction coefficients optimised from intraoperative observations	Prospective, n = 13	[84]
Other	CT-guided microcoil implantation	Retrospective, n = 40	[85]
Other	Near-infrared fluorescence imaging	Case series, n = 50	[86]
Lung	CT-guided preoperative marking with mixture of indigo carmine and lipiodol; intraoperative detection via visual dye pigmentation or fluoroscopy for radiopaque component	Retrospective, n = 157	[87]
Other	Combined intraoperative portable large field-of-view gamma camera imaging with handheld gamma detection probe for real-time tumour localisation after preoperative 111In-pentetreotide injection	Prospective, n = 5	[88]
Breast	Systemically administered ICG-p28 fluorescent probe (0.5 mg/kg IV) with 24-h tumour accumulation period before intraoperative NIR fluorescence-guided surgery using PDE imaging system	Preclinical, n = 8	[89]
Lung	Porphysome nanoparticle-mediated fluorescence imaging using scanning fiber endoscope (transbronchial) and porphysome-specific thoracoscope (transpleural) for NIR fluorescence detection	Preclinical, n = 4	[90]
Lung	Electromagnetic navigation with tracking	Prospective, n = 30	[8]
Other	Near-infrared fluorescence imaging	Prospective, n = 20	[40]

**Table 2:** Per-patient and infrastructure cost data for directly applicable technologies. NR = not reported. Cost data for transferable/adjacent technologies (RFID, magnetic seeds) derive from non-colorectal clinical contexts and are summarised narratively in Section 3.3.5 (Transferable/Adjacent Studies) rather than tabulated here, given limited comparability with colorectal-specific cost data; the underlying cost-offset model relevant to colorectal adaptation is discussed in Section 4.3. All cost figures are reported as cited in the original studies and span publications from 2007–2025; earlier figures should be interpreted as indicative rather than current.

Reference	Training/Indirect Costs	Capital/Infrastructure Cost	Per-Patient Cost	Technology
[2,29]	Minimal (1–2 cases)	USD 30,000–300,000	USD 100–300	NIR fluorescence (ICG, non-targeted)
[29]	NR	Shares NIR camera infrastructure	USD 1000–5000	NIR fluorescence (targeted probes)
[15]	Surgeon training; ongoing software licensing	NR (CT/CBCT, field generators, workstations)	NR	Electromagnetic navigation
[17]	NR	USD 200,000–400,000 (navigation) + USD 300,000–500,000 (3D C-arm)	NR	Optical stereotactic navigation (deep learning-assisted)
[24]	NR	USD 150,000–200,000	NR	Image guidance (tracked US + optical navigation)
[1]	Endoscopist expertise (existing)	None (existing endoscopy infrastructure)	USD 20–50 (dye) or USD 100–200 (clips)	Colonoscopic tattooing/metallic clip placement
[6]	IR expertise (existing)	Existing IR infrastructure	NR	CT-guided metallic clip placement
[19]	Radiation safety training, QC protocols	Gamma probes/cameras + radiation safety infrastructure	USD 100–1000 (radiopharmaceutical) + USD 500–1500 (SPECT/CT)	Radioguided surgery
[9,10]	NR	NR (prototype stage)	NR (prototype stage)	Inductive proximity sensors

**Table 3:** Methodological quality appraisal of randomised controlled trials included in this review, evaluated using the Cochrane RoB 2 tool (D1 = randomisation process; D2 = deviations from intended interventions; D3 = missing outcome data; D4 = measurement of the outcome; D5 = selection of the reported result).

Study	D1: Randomisation	D2: Deviations	D3: Missing Data	D4: Outcome Measurement	D5: Reported Result	Overall
[15]	Low risk	Some concerns	Some concerns	Low risk	Some concerns	Some concerns
[39]	Low risk	Some concerns	Low risk	Some concerns	Some concerns	Some concerns
[35]	Low risk	Some concerns	Low risk	Low risk	Low risk	Low risk

**Table 4:** Methodological quality appraisal of non-randomised studies included in this review, evaluated using the ROBINS-I tool (D1 = bias due to confounding; D2 = bias in selection of participants; D3 = bias in classification of interventions; D4 = bias due to deviations from intended interventions; D5 = bias due to missing data; D6 = bias in measurement of outcomes; D7 = bias in selection of the reported result).

Study	D1: Confounding	D2: Selection	D3: Classification	D4: Deviations	D5: Missing Data	D6: Outcomes	D7: Reporting	Overall
[2]	Moderate	Low	Low	Low	Low	Moderate	Low	Moderate
[1]	Serious	Moderate	Low	Moderate	Low	Low	Low	Serious
[18]	Low	Low	Low	Moderate	Low	Low	Low	Low
[24]	Low	Low	Low	Moderate	Low	Low	Low	Low
[25]	Serious	Moderate	Moderate	Moderate	Low	Low	Low	Serious
[33]	Low	Low	Low	Low	Low	Low	Low	Low
[42]	Serious	Serious	Low	Moderate	Low	Low	Low	Serious
[12]	Low	Low	Low	Low	Low	Low	Low	Low
[7]	Serious	Moderate	Low	Moderate	Low	Low	Low	Serious
[11]	Low	Low	Low	Low	Low	Low	Low	Low

**Table 5:** Evidence maturity characterisation using the GRADE framework for primary detection outcomes across the four main technology groups.

Technology	Key Outcome	No. Studies	Study Design	Risk of Bias	Inconsistency	Indirectness	Imprecision	Publication Bias	Evidence Maturity
ICG/NIR Fluorescence	Detection rate	10	Observational	Serious	Serious	Not serious	Serious	Suspected	Very Low
EM Navigation	Complete target removal	3	1 RCT + 2 prospective	Moderate	Serious	Not serious	Not serious	Unlikely	Low
RFID Localisation	Successful tag retrieval	4	Observational	Serious	Serious	Very serious	Not serious	Suspected	Very Low
Magnetic Seeds	Successful localisation	3	1 RCT + 2 observational	Moderate	Not serious	Very serious	Serious	Suspected	Very Low

## Data Availability

The data supporting the findings of this scoping review are contained within the article and its Supplementary Materials. Supplementary Table S1 provides the complete data extraction matrix for all 89 included studies. Supplementary Table S2 provides the technology-level evidence synthesis. Supplementary File S3 provides the completed PRISMA-ScR checklist. No new datasets were generated. All primary data sources are publicly available peer-reviewed publications cited in the reference list.

## Supplementary Materials

The following supporting information can be downloaded at: https://www.mdpi.com/article/10.3390/diagnostics16131952/s1, Table S1: Complete data extraction matrix for 89 studies included in systematic review: Tumour Localisation Technologies in Colorectal Cancer Surgery: A Systematic Review of Marking and Detection Methods; Table S2: Technology-Level Evidence Synthesis Systematic Review: Tumour Localisation Technologies in Colorectal Cancer Surgery: A Systematic Review of Marking and Detection Methods; File S3: the completed PRISMA-ScR checklist.

## Appendix A. Database Search Strategies

Complete search strategies used for this scoping review. Searches were conducted in December 2025 covering publications from 2000 to 2025.

### Appendix A.1. PubMed/MEDLINE Search Strategy

**PubMed/MEDLINE**	**Database**
December 2025	Date of Search
2000–2025	Date Range
English	Language Filter
1185	Results Retrieved

Search Query:

((colorectal[Title/Abstract] OR colon[Title/Abstract] OR rectal[Title/Abstract] OR rectum[Title/Abstract]) AND (tumor*[Title/Abstract] OR tumour*[Title/Abstract] OR lesion*[Title/Abstract] OR polyp*[Title/Abstract] OR cancer*[Title/Abstract] OR metastas*[Title/Abstract]) AND (localization[Title/Abstract] OR localisation[Title/Abstract] OR detection[Title/Abstract] OR identification[Title/Abstract] OR marking[Title/Abstract] OR navigation[Title/Abstract] OR guidance[Title/Abstract]) AND (laparoscop*[Title/Abstract] OR “minimally invasive”[Title/Abstract] OR endoscop*[Title/Abstract]) AND (RFID[Title/Abstract] OR “radio-frequency”[Title/Abstract] OR electromagnetic[Title/Abstract] OR magnetic[Title/Abstract] OR fluorescence[Title/Abstract] OR ICG[Title/Abstract] OR “indocyanine green”[Title/Abstract] OR “near-infrared”[Title/Abstract] OR NIR[Title/Abstract] OR optical[Title/Abstract] OR radioguided[Title/Abstract] OR tattoo*[Title/Abstract] OR “india ink”[Title/Abstract] OR clip*[Title/Abstract] OR marker*[Title/Abstract] OR sensor*[Title/Abstract] OR tracking[Title/Abstract] OR “image-guided”[Title/Abstract]))

### Appendix A.2. Scopus Search Strategy

**Scopus**	**Database**
December 2025	Date of Search
2000–2025	Date Range
English	Language Filter
Article, Review, Conference Paper	Document Types
3213	Results Retrieved

Search Query:

TITLE-ABS-KEY((colorectal OR colon OR rectal OR rectum) AND (tumor* OR tumour* OR lesion* OR polyp* OR cancer* OR metastas*) AND (localization OR localisation OR detection OR identification OR marking OR navigation OR guidance) AND (laparoscop* OR “minimally invasive” OR endoscop*) AND (rfid OR “radio-frequency” OR “radiofrequency” OR electromagnetic OR magnetic OR fluorescence OR icg OR “indocyanine green” OR “near-infrared” OR nir OR optical OR radioguided OR tattoo* OR “india ink” OR clip* OR marker* OR sensor* OR tracking OR “image-guided” OR “image guided”))

### Appendix A.3. Web of Science Search Strategy

**Web of Science Core Collection**	**Database**
December 2025	Date of Search
2000–2025	Date Range
English	Language Filter
Article, Review Article, Proceedings Paper	Document Types
510	Results Retrieved

Search Query:

TS=((colorectal OR colon OR rectal OR rectum) AND (tumor* OR tumour* OR lesion* OR polyp* OR cancer* OR metastas*) AND (localization OR localisation OR detection OR identification OR marking OR navigation OR guidance) AND (laparoscop* OR “minimally invasive” OR endoscop*) AND (RFID OR “radio-frequency” OR radiofrequency OR electromagnetic OR magnetic OR fluorescence OR ICG OR “indocyanine green” OR “near-infrared” OR NIR OR optical OR radioguided OR tattoo* OR “india ink” OR clip* OR marker* OR sensor* OR tracking OR “image-guided” OR “image guided”))

Notes on Search Strategy

- All searches were conducted without MeSH terms or subject headings to ensure comprehensive coverage across databases with different indexing systems.
- Wildcard symbols (*) were used for word truncation to capture variant word endings (e.g., tumor*/tumour*, laparoscop*).
- Both American and British spelling variants were included where applicable (e.g., localization/localisation, tumor/tumour).
- The search strategy was designed to be sensitive, prioritising comprehensive retrieval over precision to minimise risk of missing relevant studies.
- Reference list screening of included studies was performed; no additional eligible studies were identified.
